# Bayesian Test for Colocalisation between Pairs of Genetic Association Studies Using Summary Statistics

**DOI:** 10.1371/journal.pgen.1004383

**Published:** 2014-05-15

**Authors:** Claudia Giambartolomei, Damjan Vukcevic, Eric E. Schadt, Lude Franke, Aroon D. Hingorani, Chris Wallace, Vincent Plagnol

**Affiliations:** 1 UCL Genetics Institute, University College London (UCL), London, United Kingdom; 2 Murdoch Childrens Research Institute, Royal Children's Hospital, Melbourne, Australia; 3 Department of Genetics and Genomics Sciences, Mount Sinai School of Medicine, New York, New York, United States of America; 4 Department of Genetics, University Medical Center Groningen, University of Groningen, Groningen, The Netherlands; 5 Institute of Cardiovascular Science, University College London, London, United Kingdom; 6 JDRF/Wellcome Trust Diabetes and Inflammation Laboratory, Cambridge, Institute for Medical Research, Department of Medical Genetics, NIHR, Cambridge Biomedical Research Centre, University of Cambridge, Addenbrooke's Hospital, Cambridge, United Kingdom; Dartmouth College, United States of America

## Abstract

Genetic association studies, in particular the genome-wide association study (GWAS) design, have provided a wealth of novel insights into the aetiology of a wide range of human diseases and traits, in particular cardiovascular diseases and lipid biomarkers. The next challenge consists of understanding the molecular basis of these associations. The integration of multiple association datasets, including gene expression datasets, can contribute to this goal. We have developed a novel statistical methodology to assess whether two association signals are consistent with a shared causal variant. An application is the integration of disease scans with expression quantitative trait locus (eQTL) studies, but any pair of GWAS datasets can be integrated in this framework. We demonstrate the value of the approach by re-analysing a gene expression dataset in 966 liver samples with a published meta-analysis of lipid traits including >100,000 individuals of European ancestry. Combining all lipid biomarkers, our re-analysis supported 26 out of 38 reported colocalisation results with eQTLs and identified 14 new colocalisation results, hence highlighting the value of a formal statistical test. In three cases of reported eQTL-lipid pairs (*SYPL2*, *IFT172*, *TBKBP1*) for which our analysis suggests that the eQTL pattern is not consistent with the lipid association, we identify alternative colocalisation results with *SORT1*, *GCKR*, and *KPNB1*, indicating that these genes are more likely to be causal in these genomic intervals. A key feature of the method is the ability to derive the output statistics from single SNP summary statistics, hence making it possible to perform systematic meta-analysis type comparisons across multiple GWAS datasets (implemented online at http://coloc.cs.ucl.ac.uk/coloc/). Our methodology provides information about candidate causal genes in associated intervals and has direct implications for the understanding of complex diseases as well as the design of drugs to target disease pathways.

## Author Summary

Genome-wide association studies (GWAS) have found a large number of genetic regions (“loci”) affecting clinical end-points and phenotypes, many outside coding intervals. One approach to understanding the biological basis of these associations has been to explore whether GWAS signals from intermediate cellular phenotypes, in particular gene expression, are located in the same loci (“colocalise”) and are potentially mediating the disease signals. However, it is not clear how to assess whether the same variants are responsible for the two GWAS signals or whether it is distinct causal variants close to each other. In this paper, we describe a statistical method that can use simply single variant summary statistics to test for colocalisation of GWAS signals. We describe one application of our method to a meta-analysis of blood lipids and liver expression, although any two datasets resulting from association studies can be used. Our method is able to detect the subset of GWAS signals explained by regulatory effects and identify candidate genes affected by the same GWAS variants. As summary GWAS data are increasingly available, applications of colocalisation methods to integrate the findings will be essential for functional follow-up, and will also be particularly useful to identify tissue specific signals in eQTL datasets.

## Introduction

In the last decade, hundreds of genomic loci affecting complex diseases and disease relevant intermediate phenotypes have been found and robustly replicated using genome-wide association studies (GWAS, [1]). At the same time, gene expression measurements derived from microarray [2] or RNA sequencing [3] studies have been used extensively as an outcome trait for the GWAS design. Such studies are usually referred to as expression quantitative trait locus (eQTL) analysis. While GWAS datasets have provided a steady flow of positive and replicable results, the interpretation of these findings, and in particular the identification of underlying molecular mechanisms, has proven to be challenging. Integrating molecular level data and other disease relevant intermediate phenotypes with GWAS results is the natural step forward in order to understand the biological relevance of these results. This strategy has been explored before and allowed the identification of the genes and regulatory variations that are important for several diseases (reviewed in [4]).

In this context, a natural question to ask is whether two independent association signals at the same locus, typically generated by two GWAS studies, are consistent with a shared causal variant. If the answer is positive, we refer to this situation as colocalised traits, and the probability that both traits share a causal mechanism is greatly increased. A typical example involves an eQTL study and a disease association result, which points to the causal gene and the tissue in which the effect is mediated [5]–[7]. In fact, looking for overlaps between complex trait-associated variants and eQTL variants has been successfully used as evidence of a common causal molecular mechanism (e.g., [5], [8]). The same questions can also be considered between pairs of eQTLs [9], [10], or pairs of diseases [11].

However, identifying the traits that share a common association signal is not a trivial statistical task. Visual comparison of overlaps of association signals with an expression dataset is a step in this direction (using for example Sanger tool Genevar http://www.sanger.ac.uk/resources/software/genevar/), but the abundance of eQTLs in the human genome and across different tissues makes an accidental overlap between these signals very likely [2]. Therefore visual comparison is not enough to make inferences about causality and formal statistical tests must be used to address this question.

Nica et al. [5] proposed a methodology to rank the SNPs with an influence on two traits based on the residual association conditional on the most associated SNP. By comparing the GWAS SNP score with all other SNPs in the associated region, this method accounts for the local LD structure. However, this is not a formal test of a null hypothesis for, or against, colocalisation at the locus of interest. A formal test of colocalisation has been developed in a regression framework. This is based on testing a null hypothesis of proportionality of regression coefficients for two traits across any set of SNPs, an assumption which should hold whenever they share causal variant(s) [12], [13]. No assumption is made about the number of causal variants, although the method does assume that in the case of multiple causal variants, all are shared. Both the ranking method and proportionality testing share the drawback of having to specify a subset of SNPs to base the test on, and Wallace [14] shows that this step can generate significant biases. The main sources of bias are overestimation of effect sizes at selected SNPs (termed “Winner's curse”), and the fact that, owing to random fluctuations, the causal variant may not always be the most strongly associated one. These factors lead to rejection of colocalisation in situations where the causal SNP is in fact shared. Although this can be overcome in the case of proportionality testing by averaging over the uncertainty associated with the best SNP models [14], perhaps the greatest limitation is the requirement for individual level genotype data, which are rarely available for large scale eQTL datasets.

The success of GWAS meta-analyses has shown that there is considerable benefit in being able to derive association tests on the basis of summary statistics. With these advantages in mind, He et al. [7] developed a statistical test to match the pattern of gene expression with a GWAS dataset. This approach, coded in the software Sherlock, can accommodate p-values as input. However, their hypothesis of interest differs from the question of colocalisation, with the focus of the method being on genome-wide convergence of signals, assuming an abundance of trans eQTLs. In particular, SNPs that are not associated with gene expression do not contribute to the test statistic. Such variants can provide strong evidence against colocalisation if they are strongly associated with the GWAS outcome.

These limitations motivate the development of novel methodologies to test for colocalisation between pairs of traits. Here, we derive a novel Bayesian statistical test for colocalisation that addresses many of the shortcomings of existing tools. Our analysis focuses on a single genomic region at a time, with a major focus on interpreting the pattern of LD at that locus.

Our underlying model is closely related to the approach developed by Flutre et al. [10], which considers the different but related problem of maximising the power to discover eQTLs in expression datasets of multiple tissues. A key feature of our approach is that it only requires single SNP p-values and their minor allele frequencies (MAFs), or estimated allelic effect and standard error, combined with closed form analytical results that enable quick comparisons, even at the genome-wide scale. Our Bayesian procedure provides intuitive posterior probabilities that can be easily interpreted. A main application of our method is the systematic comparison between a new GWAS dataset and a large catalogue of association studies in order to identify novel shared mechanisms. We demonstrate the value of the method by re-analysing a large scale meta-analysis of blood lipids [15] in combination with a gene expression study in 966 liver samples [16].

## Results

### Overview of the method

We consider a situation where two traits have been measured in two distinct datasets of unrelated individuals. We assume that samples are drawn from the same ethnic group, i.e. allele frequencies and pattern of linkage disequilibrium (LD) are identical in both populations. For each of the two samples, we consider for each variant a linear trend model between the outcome phenotypes *Y* and the genotypes *X* (or a log-odds generalised linear model if one of the two outcome phenotypes *Y* is binary):
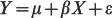
We are interested in a situation where single variant association p-values and MAFs, or estimated regression coefficients 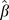 and their estimated precisions 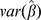, are available for both datasets at *Q* variants, typically SNPs but also indels. We make two additional assumptions and discuss later in this paper how these can be relaxed. Firstly, that the causal variant is included in the set of *Q* variants, either directly typed or well imputed [17]–[19]. Secondly, that at most one association is present for each trait in the genomic region of interest. We are interested in exploring whether the data support a shared causal variant for both traits. While the method is fully applicable to a case-control outcome, we consider two quantitative traits in this initial description.

**Figure 1:**
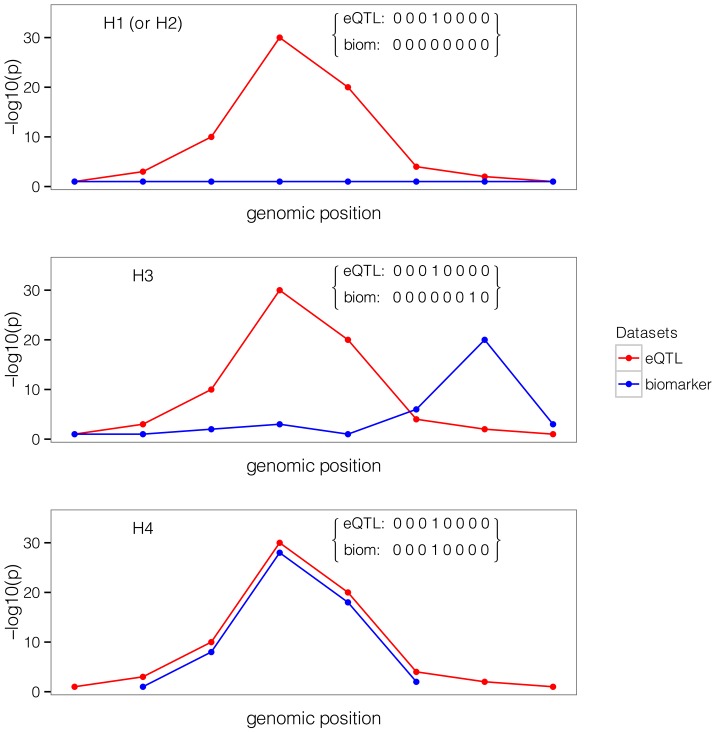
Example of one configuration under different hypotheses. A configuration is represented by one binary vector for each trait of (0,1) values of length n = 8, the number of shared variants in a region. The value of 1 means that the variant is causally involved in disease, 0 that it is not. The first plot shows the case where only one dataset shows an association. The second plot shows that the causal SNP is different for the biomarker dataset compared to the expression dataset. The third plot shows the configuration where the single causal variant is the fourth one.

SNP causality in a region of *Q* variants can be summarised for each trait using a vector of length *Q* of (0, 1) values, where 1 means that the variant is causally associated with the trait of interest and at most one entry is non-zero. A schematic illustration of this framework is provided in Figure 1 in a region that contains 8 SNPs. Each possible pair of vectors (for traits 1 and 2, which we refer to as “configuration”) can be assigned to one of five hypotheses:

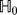
: No association with either trait

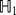
: Association with trait 1, not with trait 2

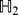
: Association with trait 2, not with trait 1

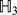
: Association with trait 1 and trait 2, two independent SNPs

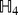
: Association with trait 1 and trait 2, one shared SNP

In this framework, the colocalisation problem can be re-formulated as assessing the support for all configurations (i.e. pairs of binary vectors) in hypothesis 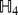.

**Figure 2:**
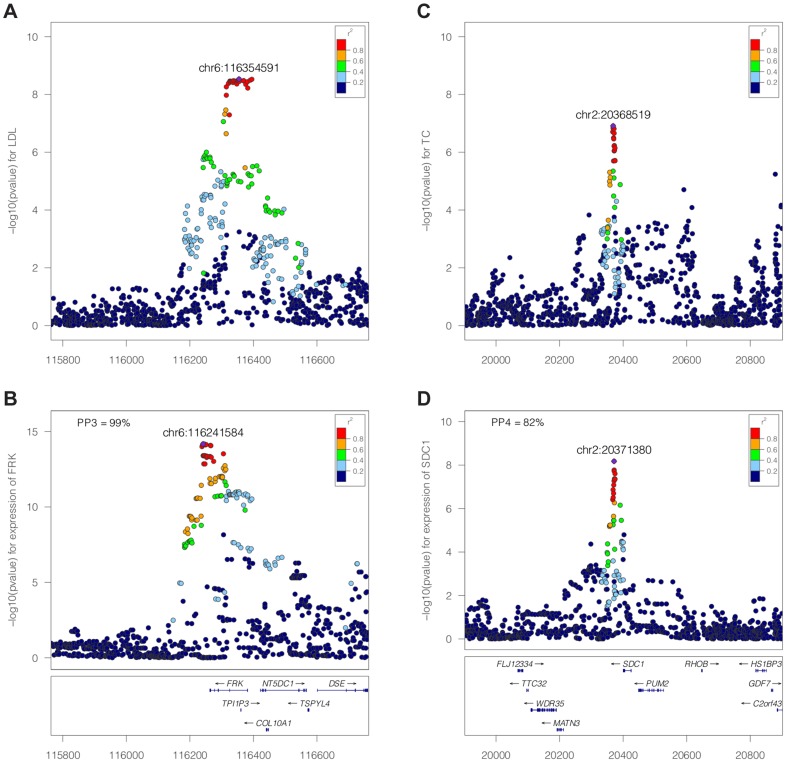
Illustration of the colocalisation results. Negative [SPACE] (A–B, FRK gene and LDL, PP3 >90%) and positive (C–D, SDC1 gene and total cholesterol, PP4 >80%) colocalisation results. −log10(p) association p-values for biomarker (top, A and C) and −log10(p) association p-values for expression (bottom, B and D) at the *FRK* (A, B) and *SDC1* locus (C, D), 1Mb range.

Our method is Bayesian in the sense that it integrates over all possible configurations. This process requires the definition of prior probabilities, which are defined at the SNP level (Methods). A probability of the data can be computed for each configuration, and these probabilities can be summed over all configurations and combined with the prior to assess the support for each hypotheses 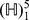. The result of this procedure is five posterior probabilities (PP0, PP1, PP2, PP3 and PP4). A large posterior probability for hypothesis 3, PP3, indicates support for two independent causal SNPs associated with each trait. In contrast, if PP4 is large, the data support a single variant affecting both traits. An illustration of the method is shown in Figure 2 for negative (Figure 2A–B, *FRK* gene and LDL, PP3 >90%) and positive (Figure 2C–D, *SDC1* gene and total cholesterol, PP4 >80%) colocalisation results.

While the method uses Approximate Bayes Factor computations (ABF, [20], and Methods), no iterative computation scheme (such as Markov Chain Monte Carlo) is required. Therefore, computations are quick and do not require any specific computing infrastructure. Precisely, the computation time behaves as 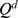, where *Q* is the number of variants in the genomic region and *d* the number distinct associations (typically *d* = 2, assuming two traits and at most one causal variant per trait).

Importantly, the use of ABF enable the computation of posterior probabilities from single variant association p-values and MAFs, although the estimated single SNP regression coefficients 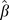 and their variances or standard errors are preferred for imputed data.

### Sample size required for colocalisation analysis

Given the well-understood requirements for large sample size for GWAS data, we used simulations to investigate the power of our approach. We generated pairs of eQTL/biomarker datasets assuming a shared causal variant. We varied two parameters: the sample size of the biomarker dataset and the proportion of the biomarker variance explained by the shared genetic variant. We set the proportion of the eQTL variance explained by the shared variant to 10% and we used the original sample size of the liver eQTL dataset described herein [16]. Text S1 contains a description of the simulation procedure.

**Figure 3:**
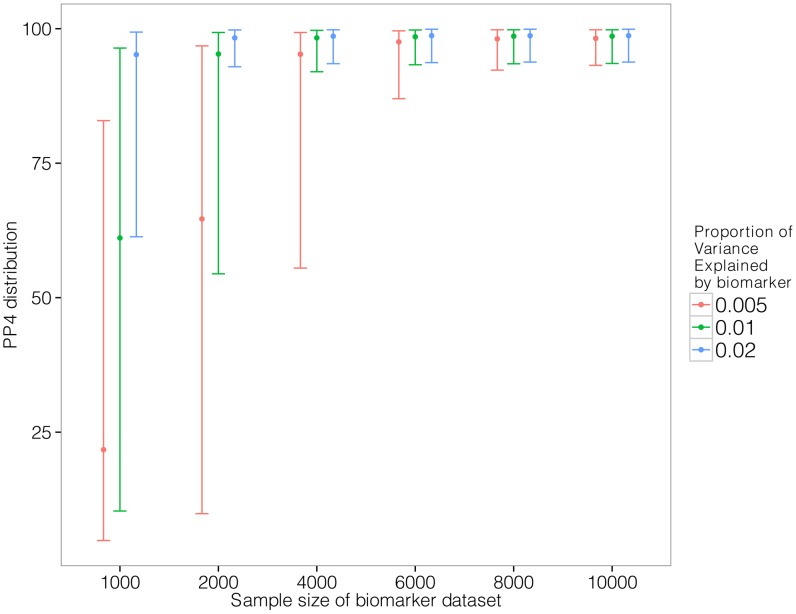
Simulation analysis with a shared causal variant between two studies. The two datasets used are one eQTL (sample size 966 samples, 10% of the variance explained by the variant) and one biomarker (such as LDL). The variance explained by the biomarker is colour coded and the x-axis shows the sample size of the biomarker study. The y axis shows the median, 10% and 90% quantile of the distribution of PP4 values (which supports a shared common variant).

Results are shown in Figure 3. We find that given a sample size of 2,000 individuals for the biomarker dataset, the causal variant needs to explain close to 2% of the variance of the biomarker to provide reliable evidence in favour of a colocalised signal (lower 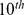 percentile for PP4 >80%).

### Consequence of limited variant density and non-additive associations

Until recently the assumption that, for a given GWAS signal, the causal variant in that interval had been genotyped was unrealistic. However, the application of imputation techniques [17]–[19] can provide genotype information about the majority of common genetic variants. Therefore, in situations where a common variant drives the GWAS signal, it is now plausible that, in imputed datasets, genotype information about this variant is available. Nevertheless, limited imputation quality can invalidate this hypothesis. This prompted us to investigate the implication of not including the causal variant in the genotype panel.

**Figure 4:**
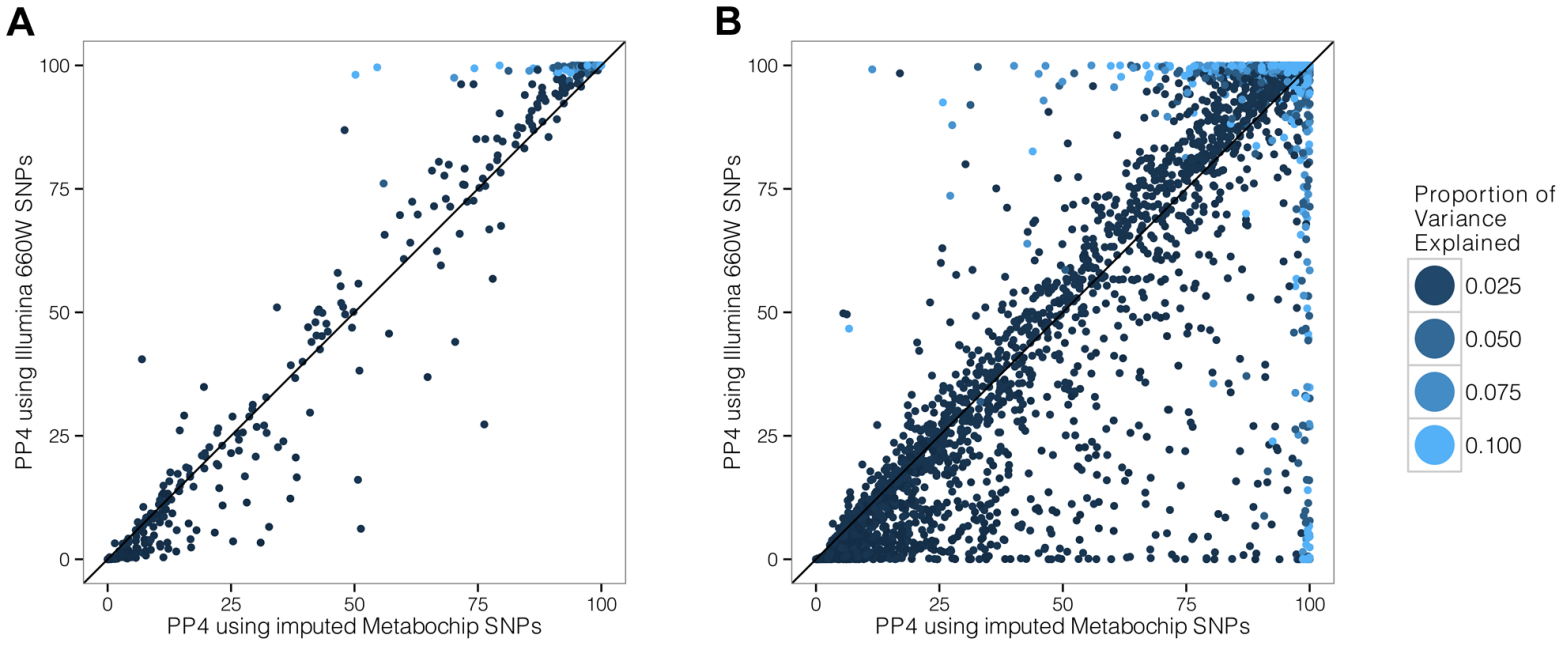
Simulation analysis with a shared causal variant between two studies. The two datasets used are one eQTL (sample size 966 samples) and one biomarker (sample size of 4,000 samples). The variance explained by the biomarker and the expression is the same and is colour coded. The x-axis shows the estimated PP4 for 1,000 simulations using data imputed from metaboChip Illumina array. The y-axis uses the same dataset restricted to variants present on the Illumina 660W genotyping array to assess the impact of a lower variant density. **A**. The causal variant is included in the Illumina 660W panel. **B**. The causal SNP not included in Illumina 660W panel.

To address this question, we used Illumina MetaboChip data and imputed the genotyped regions using the Minimac software ([19] and Methods). We then selected only the subset of variants present in the Illumina 660K genotyping array. We simulated data under the assumption of a shared causal variant, with 4,000 individuals in the biomarker dataset. We then computed the PP4 statistic with and without restricting the SNP set to the Illumina 660K Chip SNPs (Figure 4). We also considered two different scenarios, with the causal SNP included/not included in the Illumina 660W panel (Figures S1 and S2 for more exhaustive simulations).

**Figure 5:**
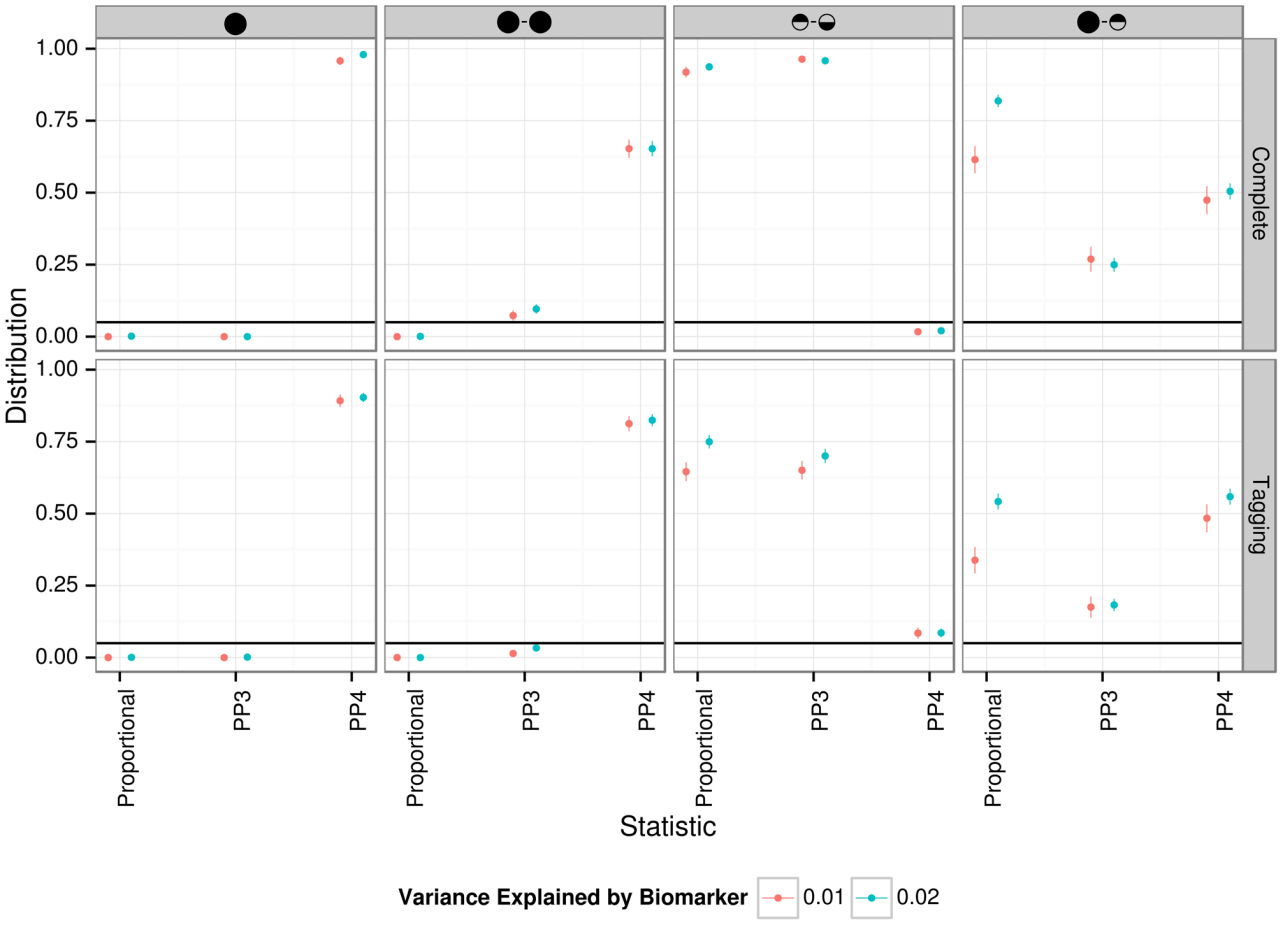
Summary of proportional and Bayesian colocalisation analysis of simulated data. Each plot shows a different scenario, the total number of causal variants in a region is indicated by number of circles in the plot titles with causal variants affecting both traits, the eQTL trait only, or the biomarker trait only, indicated by full circles, top-shaded circles and bottom-shaded circles respectively. In the top row the causal variant is typed or imputed, whereas only tag variants are typed/imputed in the bottom row. For proportional testing (under the BMA approach), we show the proportion of simulations with posterior predictive p-value <0.05 (black horizontal line) while for our Bayesian analysis we plot the proportion of simulations with the posterior probability (PP3 or PP4) of the indicated hypothesis >0.9. Error bars show 95% confidence intervals (estimated based on an average of 1,000 simulations per scenario). In all cases, for the eQTL sample size is 1,000; genetic variants explain a total of 10% of eQTL variance; for the biomarker trait, the sample size is 10,000.

Our results show that when the causal variant is directly genotyped by the low density array, the use of imputed data is not essential (Figure 4A). However, in cases where the causal variant is not typed or imputed in the low density panel, the variance of PP4 is much higher (Figure 4B). In this situation, the resulting PP4 statistic tends to decrease even though considerable variability is observed. Inspection of simulation results in Figure 5 (bottom row for tagging SNP, leftmost graph for shared causal variant) shows that while PP4 tends to be lower than for its counterpart with complete genotype data (top row, leftmost graph), PP3 remains low. This indicates that more probability is given to PP0, PP1 and PP2, which can be interpreted as a loss of power rather than misleading inference in favour of distinct variants for both traits.

Statistical power may also be affected by the mode of inheritance of the causal variant. To address this, we simulated cases under a recessive pattern of inheritance. Our results show that if the true model is recessive, but the eQTL signal is nonetheless analysed using the trend test, then we will often also successfully detect a colocalised signal (Figure S9).

### Comparison with existing colocalisation tests

We compared the behaviour of our proposed test with that of proportional colocalisation testing [12], [14] in the specific case of a biomarker dataset with 10,000 samples (Figure 5, and also Figures S3 and S4). Broadly, in the case of either a single common causal variant or two distinct causal variants, our proposed method could infer the simulated hypotheses correctly (PP4 or PP3 >0.9) with good confidence, and PP3 >0.9 slightly more often than the proportional testing p-value <0.05. A key advantage in our Bayesian approach is the ability to distinguish evidence for colocalisation (i.e. high PP4) from a lack of power (i.e. high PP0, PP1 or PP2). In both of these cases (high PP4 or high PP0/PP1/PP2), the use of the proportional approach leads to failure to reject the null even though the interpretation of these situations should differ.

It has been proposed that gene expression may be subject to both global regulatory variation which acts across multiple tissues and secondary tissue specific regulators [21]. Neither approach covers this case explicitly in its construction, but it is instructive to examine their expected behaviour. The proportional approach tends to reject a null of colocalisation, suggesting that a single distinct causal variant can be sufficient to violate the null hypothesis of proportional regression coefficients. In contrast, the Bayesian approach tends to favour the shared variant in the cases covered by our simulations (median PP4 > median PP3), and either hypotheses H3 or H4 can potentially have strong support (PP4 >0.9 in close to 50% of simulations, and PP3 >0.9 in around 25% of simulations). Of course, the ultimate goal should be to extend these tests to cover multiple causal variants, but in the meantime, it can be useful to know that a high PP4 in our proposed Bayesian analysis indicates strong support for “at least one causal variant” and that rejection of the null of proportionality of regression coefficients indicates that the two traits do not share all causal variants, not that they cannot share one.

### Dealing with several independent associations for the same trait

We have so far assumed that each trait is associated with at most one causal variant per locus. However, it is not unusual to observe two or more independent associations at a locus for a trait of interest [22]. In the presence of multiple independent associations, the assumption of a single variant per trait prompts the algorithm to consider only the strongest of these distinct association signals. Hence, the presence of additional associations that explain a smaller fraction of the variance of the trait, for example additional and independently associated rare variants, have a negligible impact on our computations.

To illustrate this situation, we simulated datasets with two causal variants: one colocalised eQTL/biomarker signal plus a secondary independent “eQTL only” signal (Figure S8). These simulations confirm that the PP4 statistic is only affected in the presence of two independent associations that explain a similar proportion of the variance of the trait (Figure S8).

The natural and statistically exact modification of our approach would compute, for each trait, Bayes factors for sets of SNPs rather than single SNPs (up to *N* SNPs jointly to accommodate for *N* distinct associations per trait). However, this approach has two drawbacks. Firstly, the interpretation of the resulting posterior probabilities is more challenging in situations where some but not all of the variants are shared across both traits. More importantly, the typical approach consists of publishing single variant summary statistics, which would prevent the use of standard summary statistics, a key feature of our approach.

**Figure 6:**
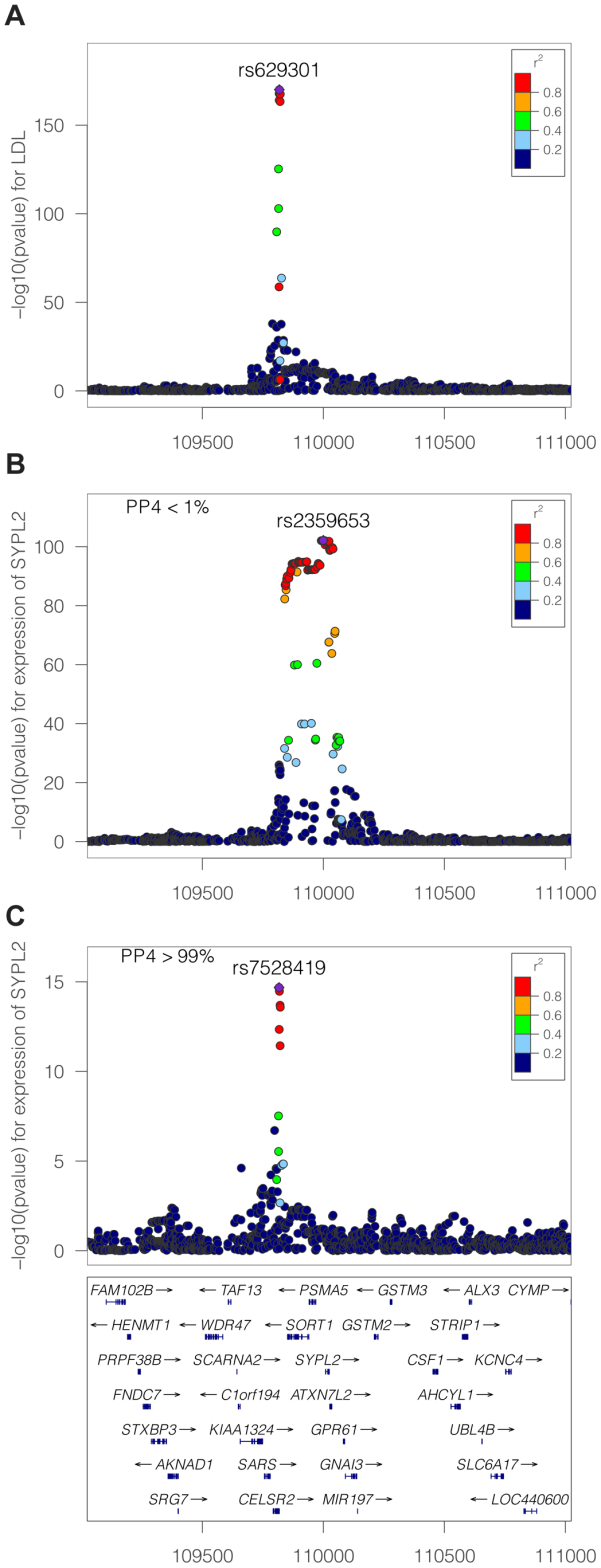
LDL association and eQTL association plots at the *SYPL2* locus. The x-axis shows the physical position on the chromosome (Mb) **A**: -log10(p) association p-values for LDL. The p-values are from the Teslovich et al published meta-analysis of >100,000 individuals. **B**: −log10(p) association p-values for *SYPL2* expression in 966 liver samples. **C**: −log10(p) association p-values for *SYPL2* expression conditional on the top eQTL associated SNP at this locus (rs2359653).

Owing to the focus of our algorithm on the strongest association signal, an alternative approach to deal with multiple associations consists of using a stepwise regression strategy, which would then reveal the secondary association signals. Our colocalisation test can then be run on using the conditional p-values. We find this approach to be the most practical and illustrate below an application for a locus that contains several independent eQTL associations (Figure 6). In situations where only single SNP summary statistics are available, the approximate conditional meta-analysis framework proposed by Visscher et al. [23] can be used to obtain conditional p-values.

### Application to a meta-analysis of blood lipids combined with a liver expression dataset

Teslovich et al. [15] reported common variants associated with plasma concentrations of low-density lipoprotein cholesterol (LDL), high-density lipoprotein cholesterol (HDL) and triglyceride (TG) levels in more than 100,000 individuals of European ancestry. They then reported the correlations between the lead SNPs at the loci they found and the expression levels of transcripts in liver. For the lipid dataset we have access only to summary statistics. The liver expression dataset used in this analysis is the same as the one used in [15]. In Teslovich et al., regions are defined within 500 kilobases of the lead SNPs, and the threshold for significance is 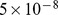. At this threshold, they found 38 SNP-to-gene eQTLs in liver (Supplementary Table 8 of [15]). Table S1 shows our results for these 38 previously reported colocalisations. A complete list of all our identified colocalisations (independently of previous reports) is provided in Tables S2, S3, S4, S5 (broken down by lipid traits). Using the coloc web server for this analysis with a PP4 >75, it took 1 minute to complete chromosome 1 and approximately 7 minutes to analyse the entire imputed genome-wide data on a laptop.

The majority of our results are consistent with the findings of Teslovich et al., with 26 out of 38 loci having PP4 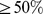. To assess the role of the prior, we varied the critical parameter 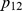, which codes for the prior probability that a variant is associated with both traits. Here we report the results using the 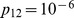. The complete list of results is provided in Table S1.

Table 1 lists the previously reported lipid-eQTL for which we find strong support *against* the colocalisation hypothesis (PP3 >75%). The LocusZoom association plots for each of these loci can be found in Figure S5. In addition to the loci listed in Table 1, we found strong evidence of distinct signals between *HLA-DQ*/*HLA-DR* and TC (Table S1) but these results must be interpreted with caution owing to the extensive polymorphism in the major histocompatibility complex region.

**Table 1:** Loci previously reported to colocalise with liver eQTL, but not supported by our analysis.

Chr	Region	Gene	Trait	Biom pval	Biom SNP	eQTL pval	eQTL SNP	Primary signal		Secondary signal*		Other genes colocalising in region (PP4 >75%)
								PP3 (%)	PP4 (%)	PP4 (%)	conditional SNP	
1	109824678∶110224737	*SYPL2*	LDL	9.7e–171	rs629301	7.1e–103	rs2359653	>99	<1	99	rs2359653	*SORT1/CELSR2/PSRC1/PSMA5*
			TC	8.0e–52	rs672569	7.1e–103	rs2359653	>99	<1	99	rs2359653	*SORT1/CELSR2/PSRC1/PSMA5*
2	27467244∶27867303	*IFT172*	TG	5.7e–133	rs1260326	1.7e–130	rs704791	>99	<1			*C2orf16/GCKR*
			TC	7.3e–27	rs1260326	1.7e–130	rs704791	>99	<1			*C2orf16/GCKR*
6	116062804∶116462863	*FRK*	LDL	2.9e–09	rs11153594	6.6e–15	rs195517	99	1			
			TC	1.7e–10	rs9488822	6.6e–15	rs195517	94	6			
17	45589357∶45989416	*TBKBP1*	LDL	1.1e–07	rs8072100	2.1e–21	rs9913503	87	9			*KPNB1*
			TC	1.8e–07	rs8072100	2.1e–21	rs9913503	92	2			*KPNB1*
19	45248464∶45648523	*APOC4*	TG	1.1e–30	rs439401	1.1e–299	19:45452692:A_AG	>99	<1	96	19:45452692:A_AG	
20	34013995:34414054	*CPNE1*	TC	3.8e–10	rs2277862	7.3e–110	rs6060524	>99	<1			

Gene/eQTL associations previously reported as having a probable shared variant but not supported by our method based on PP3 (posterior probability for distinct signal values) >75%. *Secondary signals are reported only when there is a secondary eQTL at a p-value greater than 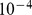. Colocalisation tests are computed using the expression data conditioned on the listed SNP. Other genes in the same region as the gene listed that colocalise using our method are reported.

For only one locus (*CEP250*), we did not find a significant eQTL signal, pointing to potential differences in bioinformatics processing and/or imputation strategy. In such a situation, both PP3 and PP4 are low and PP0, PP1 and PP2 concentrate most of the posterior distribution.

Three loci (*TMEM50A, ANGPTL3, PERLD1/PGAP3*) do not have enough evidence to strongly support either colocalisation or absence of colocalisation (Table S1) and these should remain marked as doubtful.

One of these genes, *ANGPTL3* is noteworthy. Examining this locus (Figure S6), it is clear that the pattern of association p-values is consistent between LDL and *ANGPTL3* expression. However, the extent of LD is strong, with 98 strongly associated variants. In such a situation, there is uncertainty as to whether the data support a shared causal variant for both traits, or two distincts variants for eQTL/LDL. Because the data are consistent with both scenarios, the choice of prior becomes determinant. Accordingly, PP4 drops from 91% to 49% if one uses 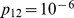 instead of 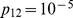.

Table 2 lists the 14 colocalised loci (15 genes) that were not reported by Teslovich et al. (or in Global Lipids Genetics Consortium [24] for the gene *NYNRIN*), but for which our method finds strong support for colocalisation (PP4 >75%). Figure S7 shows the LocusZoom plots for these colocalisation results. Eleven of these 15 genes are strong candidates for involvement in lipid metabolism and/or have been previously suggested as candidate genes: *SDC1, TGOLN2, INHBB, UBXN2B, VLDLR, VIM, CYP26A1, OGFOD1, HP, HPR, PPARA*. See Text S2 for a brief overview of the function of these genes. Four others genes have a less obvious link: *CMTM6, C6orf106, CUX2, ENSG00000259359*.

**Table 2:** Novel loci not previously reported to colocalise with liver eQTL, but colocalising based on our analysis.

Chr	Region	Gene	Trait	Biompval	BiomSNP	eQTLpval	eQTLSNP	PP3	PP4	Reference
2	20201795∶20601854	*SDC1*	TC	1.23E-07	2∶20368519	6.66E-09	2∶20371380	17	82	[41]
2	85349026∶85749085	*TGOLN2*	HDL	1.01E-07	2∶85546192	2.83E-80	2∶85553784	17	83	[42]
2	120908798∶121308857	*INHBB*	LDL	1.43E-06	2∶121305771	4.88E-21	2∶121306440	7	77	[43]
3	32322873∶32722932	*CMTM6*	TC	4.66E-06	3∶32533010	2.73E-07	3∶32523287	8	77	
6	34355095∶34755154	*C6orf106*	TC	4.68E-11	6∶34546560	4.48E-09	6∶34616322	15	85	
8	59158506∶59558565	*UBXN2B*	LDL	3.86E-09	8∶59311697	3.46E-10	8∶59331282	13	87	[44]
			TC	8.79E-13	8∶59311697	3.46E-10	8∶59331282	15	85	
9	2454062∶2854121	*VLDLR*	LDL	8.05E-06	9∶2640759	1.36E-07	9∶2640759	1	91	[45]
10	17079389∶17479448	*VIM*	TC	7.22E-07	10∶17259642	9.84E-09	10∶17260290	5	93	[46]
10	94637063∶95037122	*CYP26A1*	TG	2.38E-08	10∶94839642	3.51E-06	10∶94839724	3	95	[47]
12	111508189∶111908248	*CUX2*	HDL	4.38E-06	12∶111904371	2.81E-16	12∶111884608	2	89	
			LDL	1.73E-09	12∶111884608	2.81E-16	12∶111884608	2	98	
			TC	2.36E-11	12∶111904371	2.81E-16	12∶111884608	2	98	
15	96517293∶96917352	ENSG00000259359	HDL	8.04E-06	15∶96708291	5.50E-13	15∶96708291	2	87	
16	56310220∶56710279	*OGFOD1*	TC	3.19E-06	16∶56490549	3.36E-11	16∶56493573	7	84	[48]
16	71894416∶72310900	*HP*	LDL	1.75E-22	16∶72108093	2.15E-06	16∶72108093	1	97	[49]
			TC	3.22E-24	16∶72108093	2.15E-06	16∶72108093	1	97	
			TG	5.66E-06	16∶72108093	2.15E-06	16∶72108093	2	75	
		*HPR*	LDL	1.75E-22	16∶72108093	4.18E-08	16∶72108093	1	99	[50]
			TC	3.22E-24	16∶72108093	4.18E-08	16∶72108093	1	99	
			TG	5.66E-06	16∶72108093	4.18E-08	16∶72108093	2	89	
22	46433083∶46833138	*PPARA*	TC	3.59E-06	22∶46627603	5.96E-08	22∶46632994	10	81	[51]

Signals previously not reported as having a probable shared variant but supported by our method based on PP4 (posterior probability for a shared signal) >75% for colocalisation between the liver eQTL dataset and the Teslovich et al. meta-analysis of LDL, HDL, TG, TC, using the strict prior 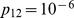. For 11 genes with strong candidate status for lipid metabolism, we list a key reference that describes their function (see Text S2 for more details of gene functions).

Three previously reported genes (*SYPL2, IFT172, TBKBP1*) which, based on our re-analysis, do not colocalise with the lipid traits, have a nearby gene with a high probability of colocalisation (respectively, *SORT1, GCKR, KPNB1*). This suggests that these genes are more likely candidates in this region. To explore the possibility that secondary signals may colocalise, we applied the stepwise regression strategy described above to deal with several independent associations at a single locus. We performed colocalisation test using eQTL results conditional on the top eQTL associated variant. Two of the loci (*SYPL2*/LDL or TC, *APOC4* and TG) showed evidence of colocalisation with expression after conditional analysis (Table 1).

An example of this stepwise procedure for the gene *SYPL2* and LDL is provided in Figure 6. We find that the top liver eQTL signal is clearly discordant with LDL association (Table 1 and Figure 6). However, conditioning on the top eQTL signal reveals a second independent association for *SYPL2* expression in liver. This secondary *SYPL2* eQTL colocalises with the LDL association (PP4 >90%, Figure 6).

### Web based resource

We developed a web site designed for integration of GWAS results using only p-values and the sample size of the datasets (http://coloc.cs.ucl.ac.uk/coloc/). The website was developed using RWUI [25]. Results include a list of potentially causal genes with the associated PP4 with their respective plots and ABF, and can be viewed either interactively or returned by email.

Researchers can request a genome-wide scan of results from a genetic association analysis, and obtain a list of genes with a high probability of mediating the GWAS signals in a particular tissue. The tool also allows visualisation of the signals within a genetic region of interest.

The database and browser currently include the possibility of investigating colocalisation with liver [15] and brain [26], [27] expression data, however the resource will soon be extended to include expression in different tissues. This method, as well as alternative approaches for colocalisation testing [12], [14], are also available with additional input options in an R package, coloc, from the Comprehensive R Archive Network (http://cran.r-project.org/web/packages/coloc).

## Discussion

We have developed a novel Bayesian statistical procedure to assess whether two association signals are colocalised. Our method is best suited for associations detected by GWAS, which are likely to reflect common, imputable, variations with small effects, or a rare variants with large effect sizes. Our aim differs from a typical fine-mapping exercise in the sense that we are not interested in knowing which variant is likely to be causal but only whether a shared causal variant is plausible. The strength of this approach lies in its speed and analytical forms, combined with the fact that it can use single variant p-values when only these are available.

Our results show that to provide an accurate answer to the colocalisation problem, high-density genotyping and/or accurate use of imputation techniques are key. The quality of the imputation is another important parameter. Indeed, while the variance of the regression coefficient can be estimated solely on the basis of the minor allele frequency for typed SNPs and sample size (and the case control ratio in the case of a binary outcome) [17], [28], this ignores the uncertainty due to imputation. Filtering out poorly imputed SNPs partially addresses this problem, with the drawback that it may exclude the causal variant(s). Hence, providing estimates of the variance of the MLE, together with the effect estimates, will result in greater accuracy. This additional option is available on the coloc package in R (http://cran.r-project.org/web/packages/coloc).

We currently assume that each genetic variant is equally likely a priori to affect gene expression or trait. A straightforward addition to our methodology would consider location specific priors for each variant, which would depend for example on the distance to the gene of interest, or the presence of functional elements in this chromosome region [29]. Our computation of the BF also assumes that, under 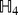, the effect sizes of the shared variant on both traits are independent. This could be modified if, for example, one compares eQTLs across different tissue types, or the same trait in two different studies. [30] has proposed a framework to deal with correlated effect sizes, and these ideas could potentially be incorporated in our colocalisation test.

Another related issue is the choice of prior probabilities for the various configurations. For the eQTL analysis, we used a 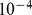 prior probability for a cis-eQTL. A more stringent threshold may be better suited for trans-eQTLs where the variants are further away from the gene under genetic control. We also used a prior probability of 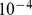 for the lipid associations. Although our knowledge about this is still lacking, this estimate has been suggested in the literature in the context of GWAS [20], [31], [32]. We assigned a prior probability of 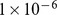 for 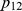, which encodes the probability that a variant affects both traits. It has been shown that SNPs associated with complex traits are more likely to be eQTLs compared to other SNPs chosen at random from GWAS platforms [33], and a higher weighting for these SNPs has been proposed when performing Bayesian association analyses [34], [35]. Also, eQTLs have been shown to be enriched for disease-associated SNPs when a disease-relevant tissue is used [9], [36]. Our sensitivity analysis for the 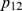 parameter showed broadly consistent results (Table S1). In cases where GWAS data are available for both traits, [10] show that it is possible to estimate these parameters from the data using a hierarchical model. This addition is a possible extension of our approach.

The interpretation of the posterior probabilities requires caution. For example, a low PP4 may not indicate evidence against colocalisation in situations where PP3 is also low. It may simply be the result of limited power, which is evidenced by high values of PP0, PP1 and/or PP2. Moreover, a high PP4 is a measure of correlation, not causality. To illustrate this, one can consider the relatively common situation where a single variant appears to affect the expression of several genes in a chromosome region (as observed, for example, in the region surrounding the *SORT1* gene). Several eQTLs will be colocalised, both between them and with the biomarker of interest. In this situation one would typically expect that a single gene is causally involved in the biomarker pathway but the colocalisation test with the biomarker will generate high PP4 values for all genes in the interval.

We show that we can use conditional p-values to deal with multiple independent associations with the same trait at one locus. While we found this solution generally effective, Wallace [14] points out that this top SNP selection for the conditional analysis can create biases, although the bias is small in the case of large samples and/or strong effects. For difficult loci with multiple associations for both traits and available genotype data, it may be more appropriate to estimate Bayes factors for sets rather than single variants in order to obtain an exact answer. This extension would avoid the issue of SNP selection for the conditional analysis.

Importantly, GWAS signals can be explained by eQTLs only when the causal variant affects the phenotype by altering the amount of mRNA produced, but not when the phenotype is affected by changing the type of protein produced, although the former seems to be the most common [33]. Furthermore, since many diseases manifest their phenotype in certain tissues exclusively [2], [21], [37], [38], colocalisation results will be dependent on the expression dataset used. In addition to identifying the causal genes, the identification of tissue specificity for the molecular effects underlying GWAS signals is a key outcome of our method. We anticipate that building a reference set of eQTL studies in multiple tissues will provide a useful check for every new GWAS dataset, pointing directly to potential candidate genes/tissue types where these effects are mediated.

While this report focuses on finding shared signals between a biomarker dataset and a liver expression dataset, we plan to utilise summary results of multiple GWAS and eQTL studies, for a variety of cell types and traits. In fact, our method can utilise summary results from any association studies. Disease/disease, (*cis* or *trans*) eQTL/disease or disease/biomarkers comparisons are all of biological interest and use the same statistical framework. We expect that the fact that the test can be based on single SNP summary statistics will be key to overcome data sharing concerns, hence enabling a large scale implementation of this tool. The increasing availability of RNA-Seq eQTL studies will further increase the opportunity to detect isoform specific eQTLs and their relevance to disease studies. Owing to the increasing availability of GWAS datasets, the systematic application of this approach will potentially provide clues into the molecular mechanisms underlying GWAS signals and the aetiology of the disorders.

## Materials and Methods

### Ethics statement

This paper re-analyses previously published datasets. All samples and patient data were handled in accordance with the policies and procedures of the participating organisations.

### Expression dataset

We used in our analysis gene expression and genotype data from 966 human liver samples. The samples were collected post-mortem or during surgical resection from unrelated European-American subjects from two different non-overlapping studies, which have been described in [16]. The cohorts were both genotyped using Illumina 650Y BeadChip array, and 39,000 expression probes were profiled using Agilent human gene expression arrays. All of the expression data has been normalised as one unit even though they were part of different studies, since high concordance between data generated using the same array platforms has been previously reported. Probe sequences were searched against the human reference genome GRCh37 from 1000 Genomes using BLASTN. Multiple probes mapping to one gene were kept in order to examine possible splicing. The probes were kept and annotated to a specific gene if they were entirely included in genes defined by Ensembl ID or by HGNC symbol using the package biomaRt in R [39]. After mapping and annotating the probes, we were left with 40,548 mapped probes covering 24,927 genes.

### Imputation of genetic data

Quality control filters were applied both before and after imputation. Before imputation, individuals with more than 10% missing genotypes were removed, and SNPs showing a missing rate greater than 10%, a deviation for HWE at a p-value less than 0.001 were dropped. After imputation, monomorphic SNPs were excluded from analyses.

To speed up the imputation process, the genome was broken into small chunks that were phased and imputed separately and then re-assembled. This was achieved using the ChunkChromosome tool (http://genome.sph.umich.edu/wiki/ChunkxChromosome), and specifying chunks of 1000 SNPs, with an overlap window of 200 SNPs on each side, which improves accuracy near the edges during the phasing step. Each chunk was phased using the program MACH1 with the number of states set to 300 and the number of rounds of MCMC set to 20 for all chunks. Phased haplotypes were used as a basis for imputation of untyped SNPs using the software Minimac with 1000 Genomes European ancestry reference haplotypes (phase1 version 3, March 2012) to impute SNPs not genotyped on the Illumina array. Variants with a MAF less than 0.001 were also excluded post-imputation. The data was then collated in probability format that can be used by the R Package snpStats [39].

### eQTL analysis

eQTL p-values, effect sizes, and standard errors were obtained by fitting a linear trend test regression between the expression of each gene and all variants 200 kilobases upstream and downstream from each probe. After filtering out the variants with MAF <0.001, monomorphic SNPs, multi-allelic SNPs (as reported in 1000 Genomes or in the Ensembl database) and variants not sufficiently well imputed (Rsq <0.3, as defined by minimac http://genome.sph.umich.edu/wiki/minimac) between both datasets, we applied our colocalisation procedure. We conducted conditional analysis on SNPs with p-values 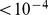 for the expression associations, and repeated the colocalisation test using expression data conditioned on the most significant SNP. The aim of this analysis is to explore whether additional signals for expression other than the main one are shared with the biomarker signal.

### Biomarker dataset

The biomarker p-values from the meta-analyses (with genomic control correction) were obtained from a publicly available repository (http://www.sph.umich.edu/csg/abecasis/public/lipids2010/).

The regional association plots for the eQTL and Biomarker datasets were created using LocusZoom [40] (http://csg.sph.umich.edu/locuszoom/).

### Posterior Computation

We call a “configuration” one possible combination of pairs of binary vectors indicating whether the variant is associated with the selected trait. We can group the configurations into five sets, 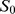, 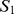, 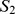, 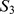, 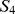, containing assignments of all SNPs *Q* to the functional role corresponding to the five hypothesis 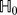, 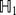, 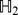, 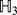, 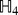. We can compute the posterior probabilities given the data for each of these 5 hypothesis by summing over the relevant configurations:
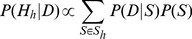
(1)where *P(S)* is the prior probability of a configuration, 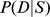 is the probability of the observed data *D* given a configuration *S*, and the sum is over all configurations *S* which are consistent with a given hypothesis 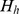, where *h* = (1,2,3,4). Thus, the probability of the data given a configuration is weighted by the prior probability of that configuration.

Next, to avoid computing the proportionality constant in Equation 1, we can reformulate the posterior probability for each hypothesis by writing this quantity as a ratio. For example, the posterior probability under hypothesis 4, dividing each of these terms by the baseline 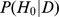, is:
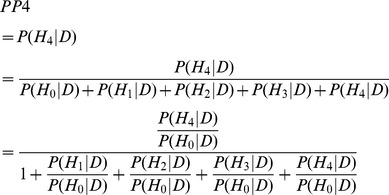
(2)


The ratios in the numerator and denominator of equation 2 are:
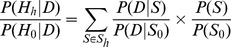
(3)The first ratio inside the sum in this equation is a Bayes Factor (BF) for each configuration, and the second ratio is the prior odds of a configuration compared with the baseline configuration 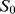. The BF can be computed for each variant from the p-value, or estimated regression coefficient 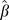 and variance of 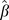, using Wakefield's method. By summing over all configurations in 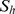 we are effectively comparing the support in the data for one alternative hypothesis versus the null hypothesis. An in-depth description of the method making use of the current assumptions can be found in Text S1.

### Bayes factor computation

A Bayes Factor for each SNP and each trait 1 and 2 was computed using the Approximate Bayes Factor (ABF, [20]). Wakefield's method yields a Bayes factor that measures relative support for a model in which the SNP is associated with the trait compared to the null model of no association.

The equation used is the following:
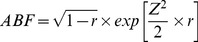
(4)where 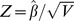 is the usual *Z* statistic and the shrinkage factor *r* is the ratio of the variance of the prior and total variance (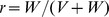). Assuming a normal distribution, the p-value of each SNP can be converted to standard one-tailed *Z*-score by using inverse normal cumulative distribution function. So for a SNP, all that it is needed are the p-values from a standard regression output, and 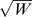, the standard deviation of the normal prior *N(0,W)* on 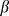. The variance of the effect estimate, *V*, can be approximated using the MAF and sample size. However for imputed data it is preferable to use the variance outputted in standard regression analysis directly in the ABF equation. For the expression dataset used here, the variance and effect estimates from the regression analysis were used for computation of ABFs (see Text S1 for more details).

### Choice of priors

Prior probabilities are assigned at the SNP level and correspond to mutually exclusive events. We assigned a prior of 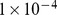 for 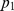 and 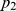, the probability that a SNP is associated with either of the two traits. Since all SNPs are assumed to have the same prior probability of association, this prior can be interpreted as an estimate for the proportion of SNPs that we expect to be associated with the trait in question. We also assigned a prior probability of 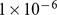 for 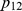, the probability that one SNP is associated with both traits. This probability can be better understood when it is re-expressed as the conditional probability of a SNP being associated with trait 2, given that it is associated with trait 1. So assigning a probability of 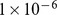 means that 1 in 100 SNPs that are associated with trait 1 is also associated with the other. As a sensitivity analysis, we ran the comparison with Teslovich et al. using two other prior probabilities for 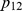, 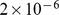 which means 1 in 50 SNPs that are associated with one trait is also associated with the other; and 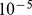 which means 1 in 10 SNPs.

To compute the ABF, we also needed to specify the standard deviation for the prior, and we set this to 0.20 for binary traits and 0.15 for quantitative traits (more details in Text S2).

## Supporting Information

Figure S1 Simulation analysis with a shared causal variant between two studies, comparing results using imputed versus not imputed data where the causal SNP is included in both the cases. The two datasets used are one eQTL (sample size 966 samples) and one biomarker, and each plot shows different sample sizes for the biomarker dataset. The variance explained by the causal variant for both the traits is colour coded. The x-axis shows the estimated PP4 for 1,000 simulations using data imputed from metaboChip Illumina array (Methods). The y-axis uses the same dataset restricted to variants present on the Illumina 660W genotyping array to assess the impact of a lower variant density. The causal variant is included in the Illumina 660W panel.
(TIF)

Figure S2 Simulation analysis with a shared causal variant between two studies, comparing results using imputed versus not imputed data where the causal SNP is not included in one of the datasets. The two datasets used are one eQTL (sample size 966 samples) and one biomarker, and each plot shows different sample sizes for the biomarker dataset. The variance explained by the causal variant for both the traits is colour coded. Column and row headings are the same as in previous figure. The causal SNP is not included in Illumina 660W panel.
(TIF)

Figure S3 The relationship between PP4 and the posterior predictive p-value (on a -log10 scale) from proportional testing. Proportional testing uses the BMA approach, integrating over all possible two SNP models. Each row shows a different scenario, the total number of causal variants in a region is indicated by number of symbols in the plot titles with the type of causal variant indicated by the symbol: full circle - affects both traits; top only - affects one trait; bottom only- affects other trait. For proportional testing, the grey vertical line indicates the threshold ppp of 0.05. Each column shows the total proportion of trait variance for the biomarker explained by all variants in a region, with variance explained spread equally over all variants. In all cases, for the eQTL trait, n = 1,000, 10% of the variance explained by the variant; for the biomarker trait, n = 10,000.
(TIF)

Figure S4 The relationship between PP4 and the posterior predictive p-value (on a -log10 scale) from proportional testing, using subset of SNPs which appear on the Illumina HumanOmniExpress genotyping array. For the eQTL trait, n = 1,000, 10% of the variance explained by the variant; for the biomarker trait, n = 10,000, 1% or 2% of the variance explained by the variant. Column and row headings are the same as in previous figure.
(TIF)

Figure S5Regional Manhattan plots corresponding to loci listed in Table 1 of main text. The plots focus on a specific region of the genome with a range of 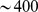 kilobases around the expression probe of the gene specified below each plot. The top plots use the -log10(p-value) from the published meta-analysis with one of the four lipid biomarkers; the bottom plots show the -log10(p-value) computed by fitting a generalised linear model with expression as dependent variable and SNP genotypes as independent variable. Each dot represents one SNP, imputed or directly typed. The value on the top of each plot shows the PP4 from the colocalisation test between the two top SNP of the expression and biomarker associations.(PDF)

Figure S6 LDL association and eQTL association plots at the *ANGPTL3* locus. The x-axis shows the physical position on the chromosome (Mb) A: −log10(p) association p-values for LDL. The p-values are from the Teslovich et al published meta-analysis of >100,000 individuals. B: −log10(p) association p-values for ANGPTL3 expression in 966 liver samples.
(TIF)

Figure S7Regional Manhattan plots corresponding to loci listed in Table 2 of main text. Row and column headers defined as in previous figure. The genomic range may be greater than 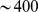 kilobases to improve visualisation of the signal.(PDF)

Figure S8 Simulation analysis with multiple shared causal variants. The first plot represents cases with only one causal variant in a region, while the following plots illustrate the behaviour of the statistic in the presence of an additional causal variant affecting the variance explained of the eQTL trait. In all scenarios, the first causal variant explains 10% of the variance of the eQTL trait. The second causal variant explains 1%, 5%, or 10% of the eQTL trait. We show the proportion of simulations with the posterior probability (PP3 or PP4) of the indicated hypothesis >0.9. Error bars show 95% confidence intervals (estimated based on an average of 1,000 simulations per scenario). In all cases, for the eQTL sample size is 1,000; for the biomarker trait, the sample size is 10,000.
(TIF)

Figure S9 Simulation analysis with a recessive shared causal variant. The two datasets used are one eQTL (sample size 966 samples, 10% of the variance explained by the variant) and one biomarker (sample size 10,000). The variance explained by the biomarker is colour coded and the shape of the dots represent the different mode of inheritance. The simulation procedure and distribution of the statistic are the same as defined in previous figure.
(TIF)

Table S1 Results using reported loci that colocalise with liver eQTL. Published results of loci correlating with both liver expression and one of the four lipid traits (Teslovich et al. Supplementary Table 8) and posterior probability of different signal (PP3) and common signal (PP4) after applying colocalisation test. Each row lists the results for one probe, and the multiple entries for the same locus and trait represent multiple probes mapping to the same locus. the columns **Biom pval** and **eQTL pval** report the lowest p-values found for the association with the trait listed and for the liver expression association respectively, with the corresponding SNP name (**Biom SNP** and **eQTL SNP**); the column **Best Causal** reports the SNP within the region with the highest posterior probability to be the true causal variant. The probabilities have been rounded to 1 significant figure.
(PDF)

Table S2eQTL/LDL colocalisation. Positive (PP4 >75%) eQTL/LDL colocalisation results between the liver eQTL dataset and the Teslovich meta-analysis using the most stringent prior for the probability that one SNP is associated with both traits, 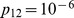. The column **Signal** includes genes that are part of overlapping regions and that colocalise at PP4 >75%; the column **Region** represents the genomic coordinates for the start and stop of the signal; in the column **Tesl**, “Y” indicates that this signal with any of the genes included has been reported to be an intermediate for any of the four lipid biomarker associations by Teslovich et al. ; the columns **Biom pval** and **eQTL pval** report the lowest p-values found for LDL association and for the expression association respectively, with the corresponding SNP name (**Biom SNP** and **eQTL SNP**); the column **Best Causal** reports the SNP within the region with the highest posterior probability to be the true causal variant. The probabilities have been rounded to 1 significant figure.(PDF)

Table S3 eQTL/HDL colocalisation. Positive (PP4 >75%) eQTL/HDL colocalisation results between the liver eQTL dataset and the Teslovich meta-analysis. Column and row headings are the same as in previous figure.
(PDF)

Table S4 eQTL/TG colocalisation. Positive (PP4 >75%) eQTL/HDL colocalisation results between the liver eQTL dataset and the Teslovich meta-analysis. Column and row headings are the same as in previous figure.
(PDF)

Table S5 eQTL/TC colocalisation. Positive (PP4 >75%) eQTL/HDL colocalisation results between the liver eQTL dataset and the Teslovich meta-analysis. Column and row headings are the same as in previous figure.
(PDF)

Text S1 Supplementary materials. Expanded methods, derivations and analyses.
(PDF)

Text S2 Overview of gene function of new colocalisation results associated with blood lipid levels and liver expression.
(PDF)
