# Supplementary figures and images for: Bayesian Test for Colocalisation between Pairs of Genetic Association Studies Using Summary Statistics
Source: PLoS Genet. 2014 May 15;10(5):e1004383. doi: 10.1371/journal.pgen.1004383 (PMC4022491; doi:10.1371/journal.pgen.1004383)

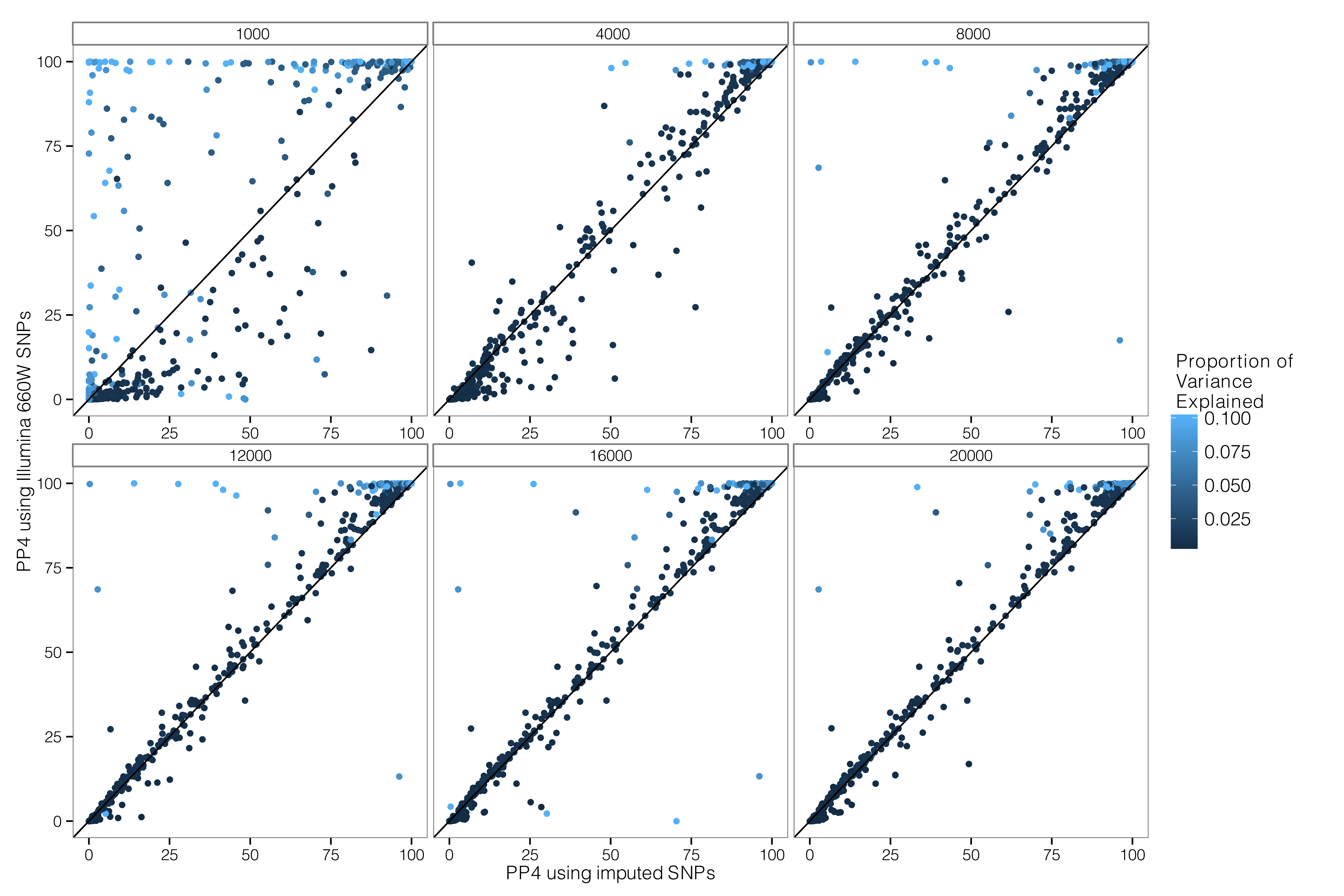

Supplement: Figure S1 — Simulation analysis with a shared causal variant between two studies, comparing results using imputed versus not imputed data where the causal SNP is included in both the cases. The two datasets used are one eQTL (sample size 966 samples) and one biomarker, and each plot shows different sample sizes for the biomarker dataset. The variance explained by the causal variant for both the traits is colour coded. The x-axis shows the estimated PP4 for 1,000 simulations using data imputed from metaboChip Illumina array (Methods). The y-axis uses the same dataset restricted to variants present on the Illumina 660W genotyping array to assess the impact of a lower variant density. The causal variant is included in the Illumina 660W panel. (TIF) [file pgen.1004383.s001.tif]

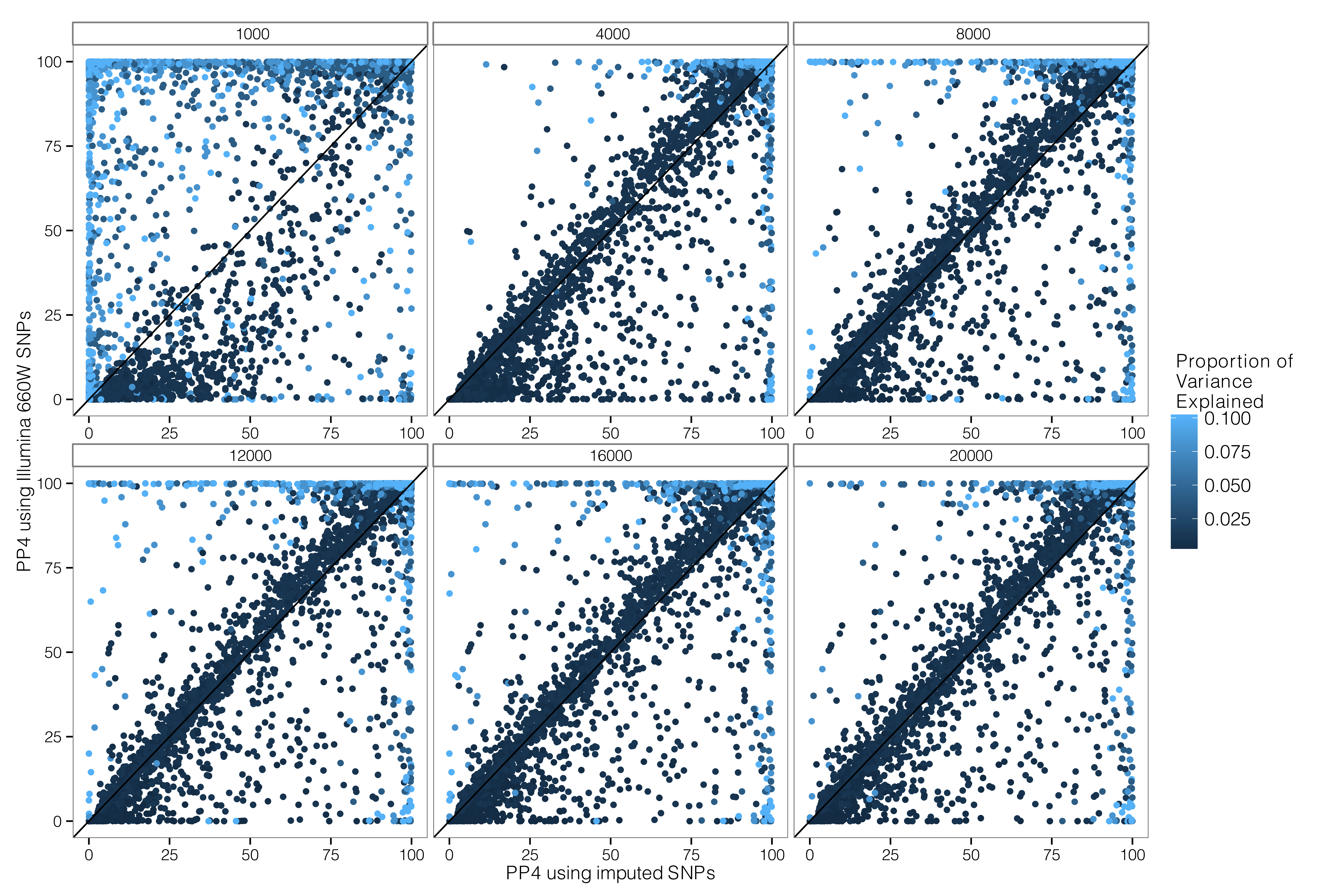

Supplement: Figure S2 — Simulation analysis with a shared causal variant between two studies, comparing results using imputed versus not imputed data where the causal SNP is not included in one of the datasets. The two datasets used are one eQTL (sample size 966 samples) and one biomarker, and each plot shows different sample sizes for the biomarker dataset. The variance explained by the causal variant for both the traits is colour coded. Column and row headings are the same as in previous figure. The causal SNP is not included in Illumina 660W panel. (TIF) [file pgen.1004383.s002.tif]

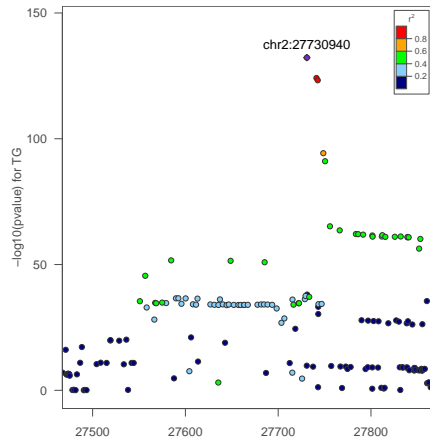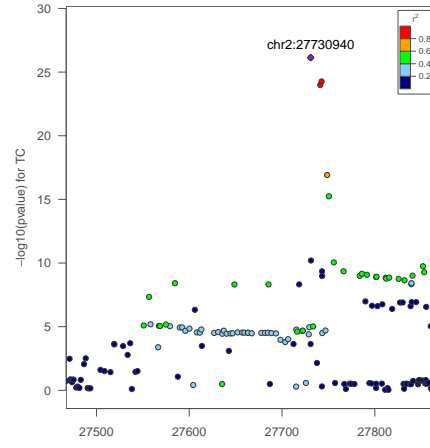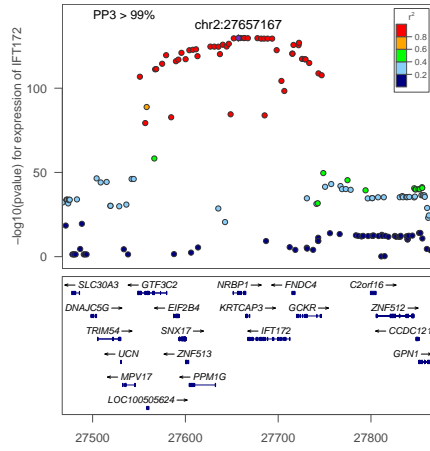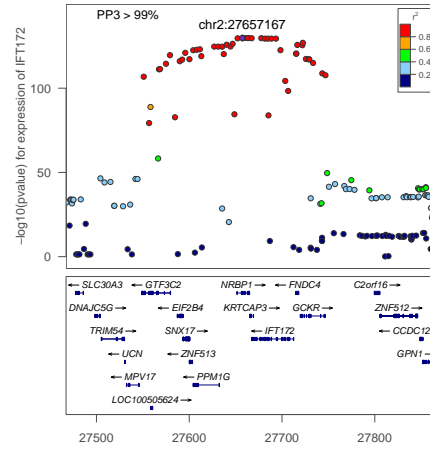

(1) IFT172/TG

(2) IFT172/TC

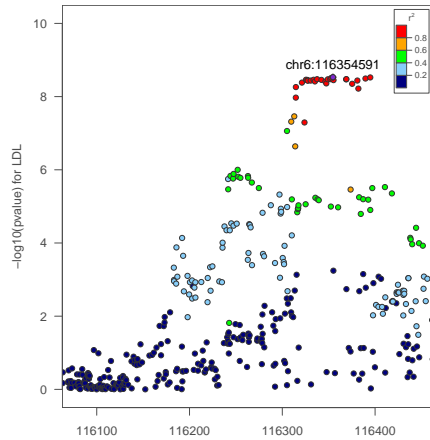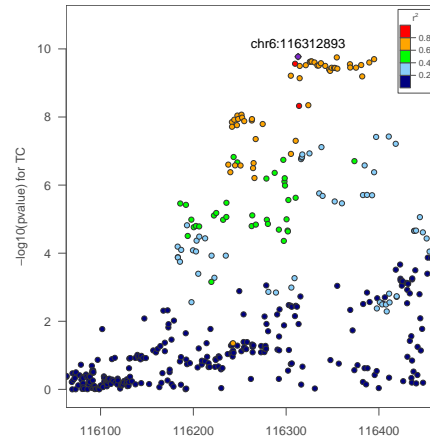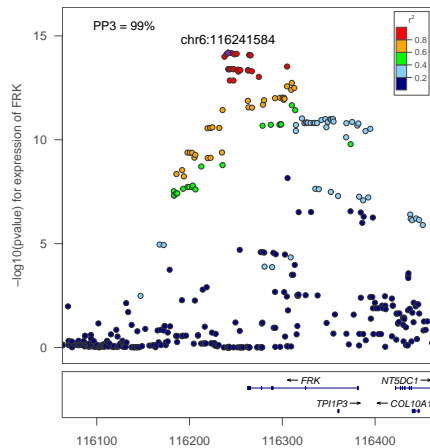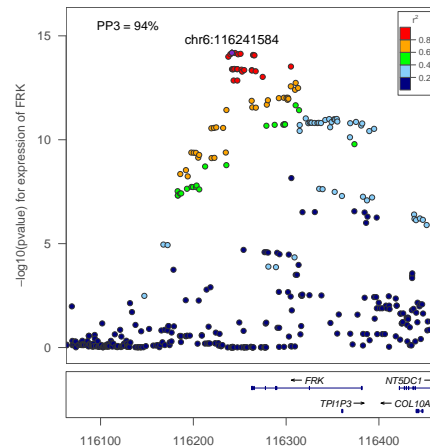

(3) FRK/LDL

(4) FRK/TC

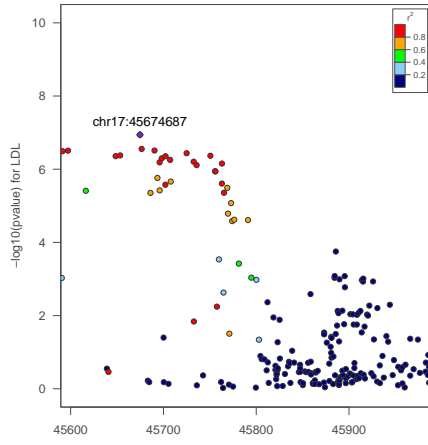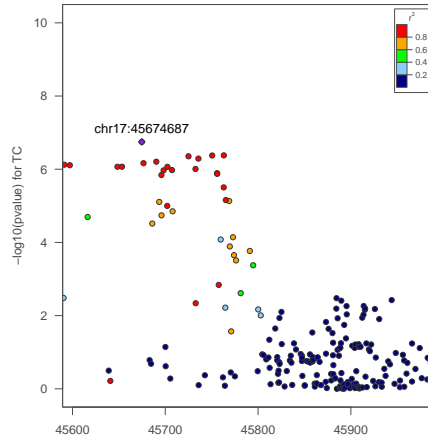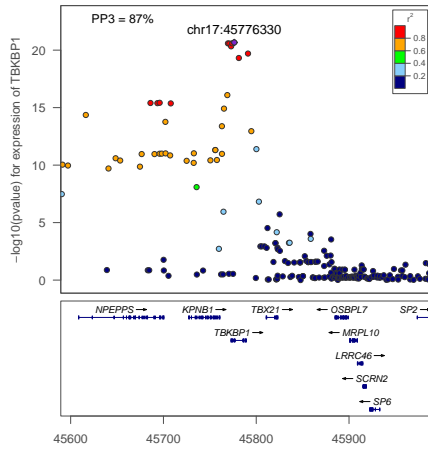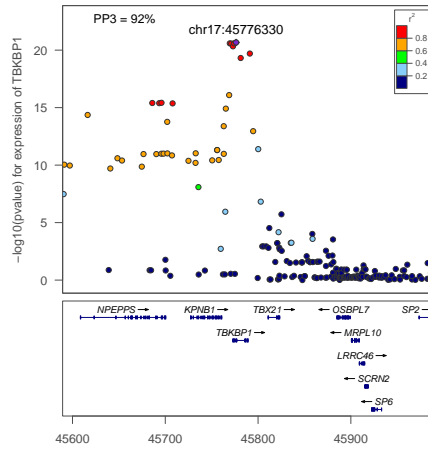

(5) TBKBP1/LDL

(6) TBKBP1/TC

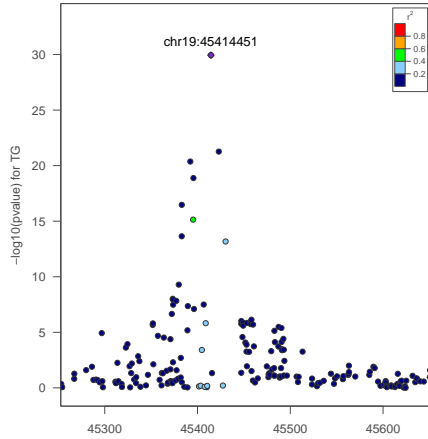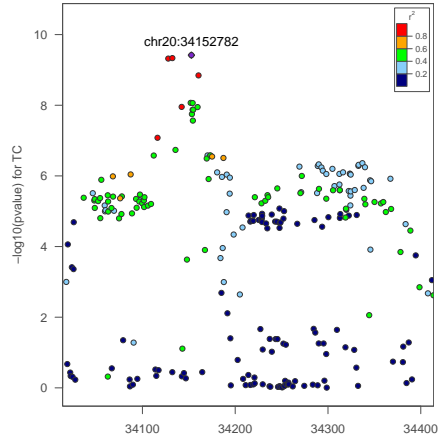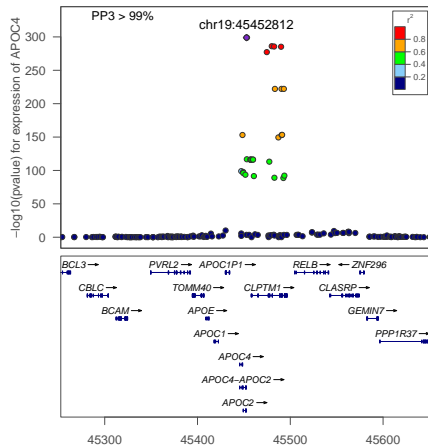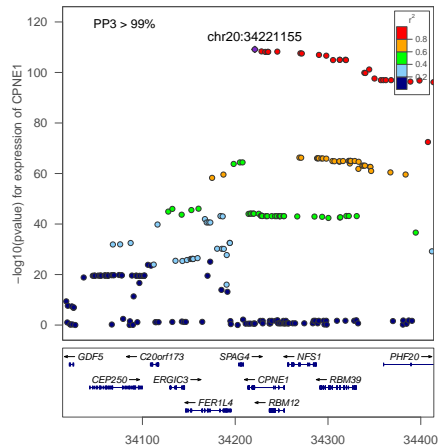

(7) APOC4/TG

(8) CPNE1/TC

Supplement: Figure S5 — Regional Manhattan plots corresponding to loci listed in Table 1 of main text. The plots focus on a specific region of the genome with a range of kilobases around the expression probe of the gene specified below each plot. The top plots use the -log10(p-value) from the published meta-analysis with one of the four lipid biomarkers; the bottom plots show the -log10(p-value) computed by fitting a generalised linear model with expression as dependent variable and SNP genotypes as independent variable. Each dot represents one SNP, imputed or directly typed. The value on the top of each plot shows the PP4 from the colocalisation test between the two top SNP of the expression and biomarker associations. (PDF) [file pgen.1004383.s005.pdf]

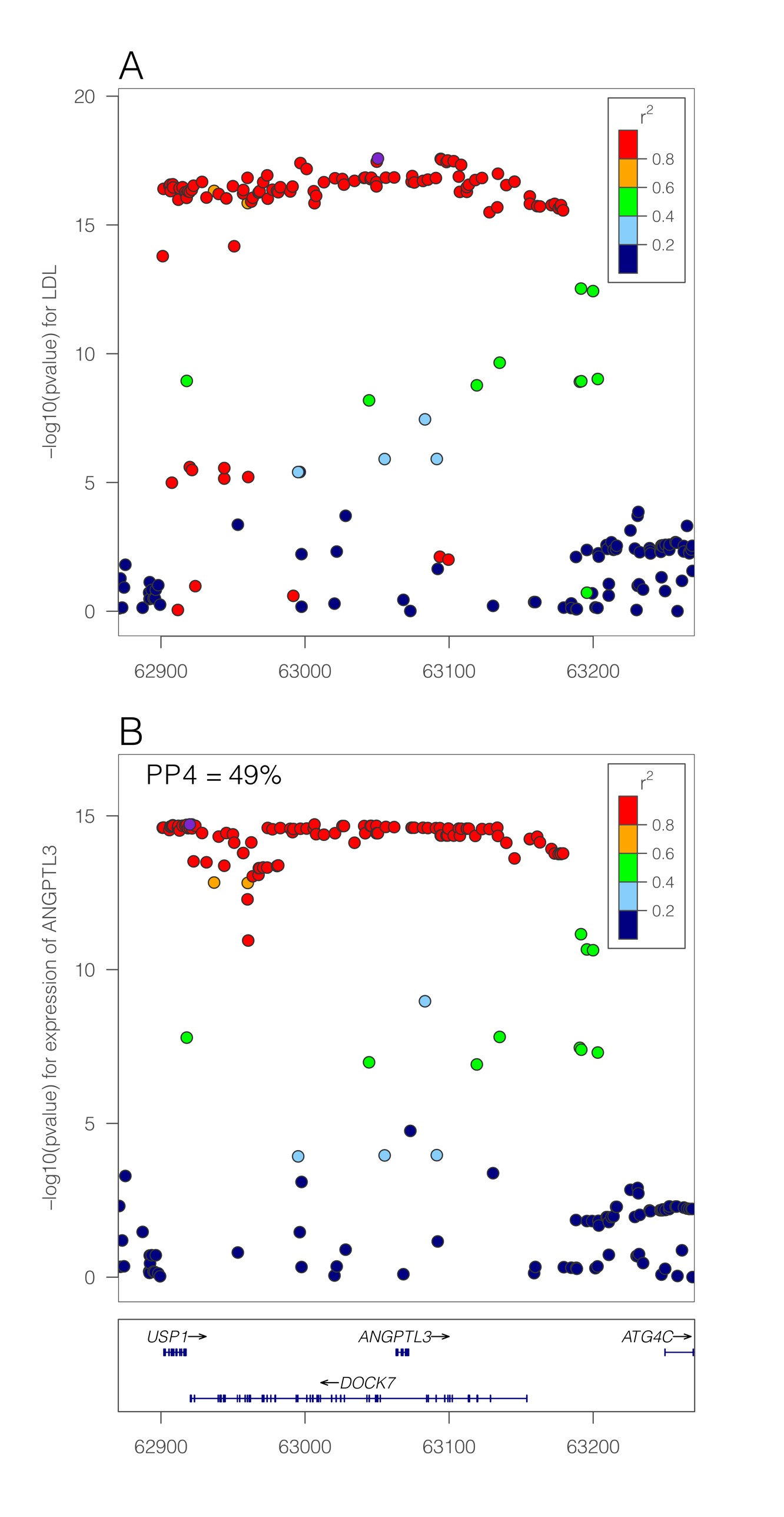

Supplement: Figure S6 — LDL association and eQTL association plots at the ANGPTL3 locus. The x-axis shows the physical position on the chromosome (Mb) A: −log10(p) association p-values for LDL. The p-values are from the Teslovich et al published meta-analysis of >100,000 individuals. B: −log10(p) association p-values for ANGPTL3 expression in 966 liver samples. (TIF) [file pgen.1004383.s006.tif]

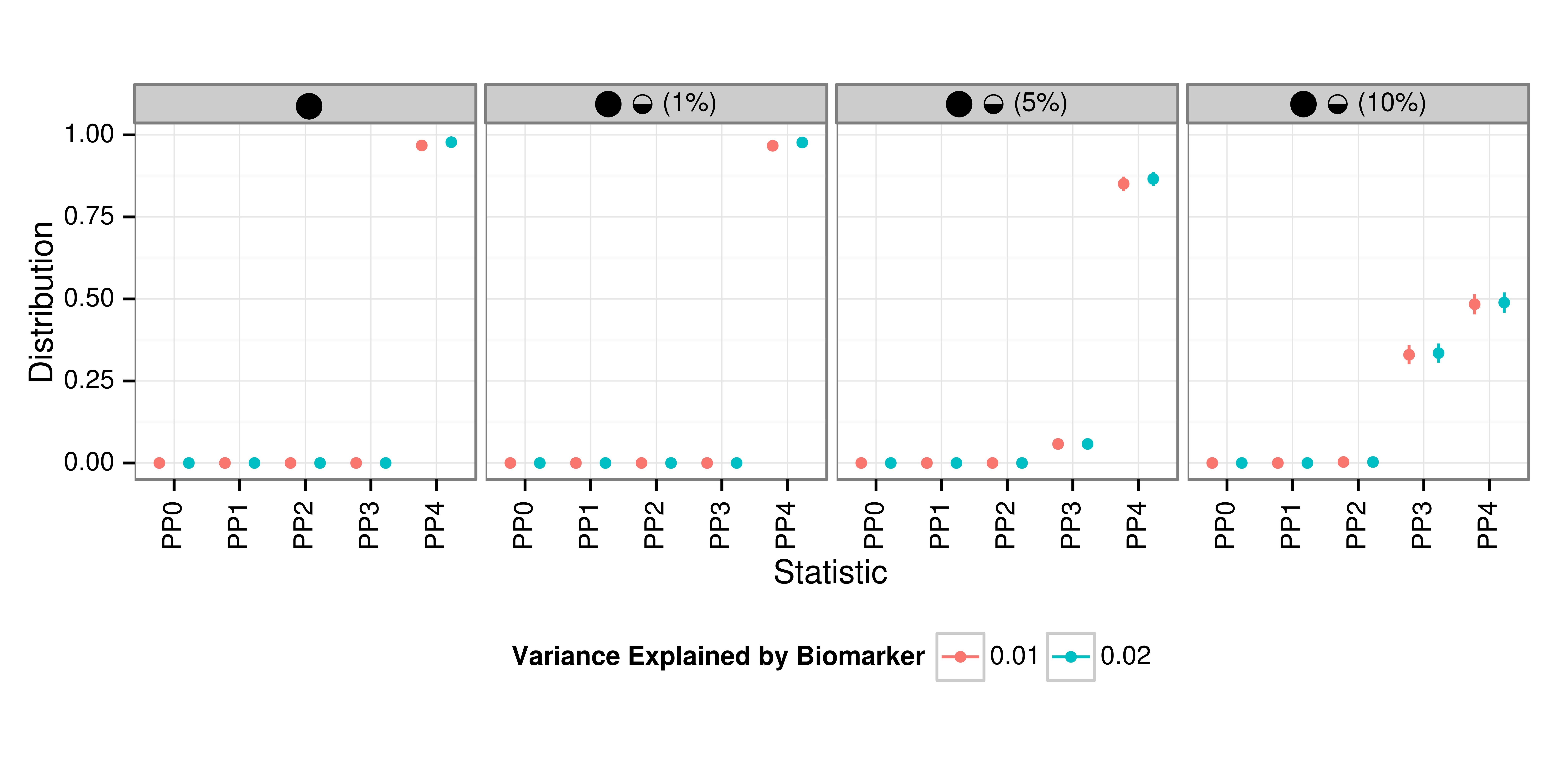

Supplement: Figure S8 — Simulation analysis with multiple shared causal variants. The first plot represents cases with only one causal variant in a region, while the following plots illustrate the behaviour of the statistic in the presence of an additional causal variant affecting the variance explained of the eQTL trait. In all scenarios, the first causal variant explains 10% of the variance of the eQTL trait. The second causal variant explains 1%, 5%, or 10% of the eQTL trait. We show the proportion of simulations with the posterior probability (PP3 or PP4) of the indicated hypothesis >0.9. Error bars show 95% confidence intervals (estimated based on an average of 1,000 simulations per scenario). In all cases, for the eQTL sample size is 1,000; for the biomarker trait, the sample size is 10,000. (TIF) [file pgen.1004383.s008.tif]
