# Supplementary material for: Bayesian Test for Colocalisation between Pairs of Genetic Association Studies Using Summary Statistics
Source: PLoS Genet. 2014 May 15;10(5):e1004383. doi: 10.1371/journal.pgen.1004383 (PMC4022491; doi:10.1371/journal.pgen.1004383)
Supplement: Figure S7 — Regional Manhattan plots corresponding to loci listed in Table 2 of main text. Row and column headers defined as in previous figure. The genomic range may be greater than kilobases to improve visualisation of the signal. (PDF) [file pgen.1004383.s007.pdf]

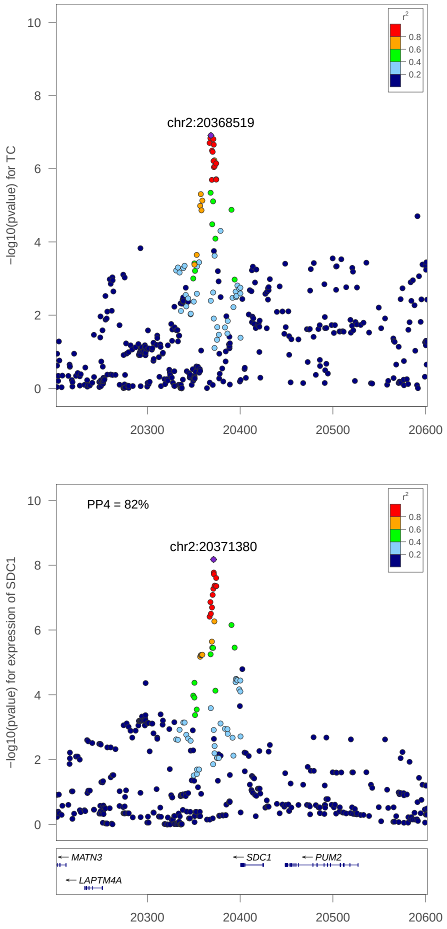

(1) SDC1/TC

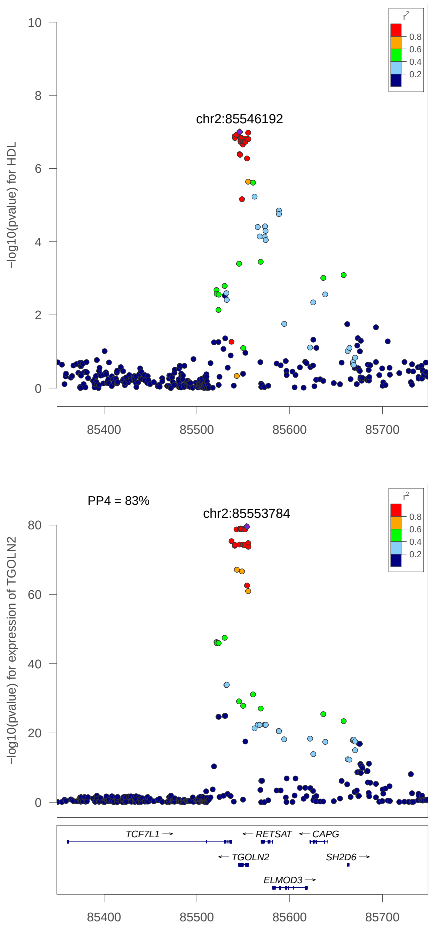

(2) TGOLN2/HDL

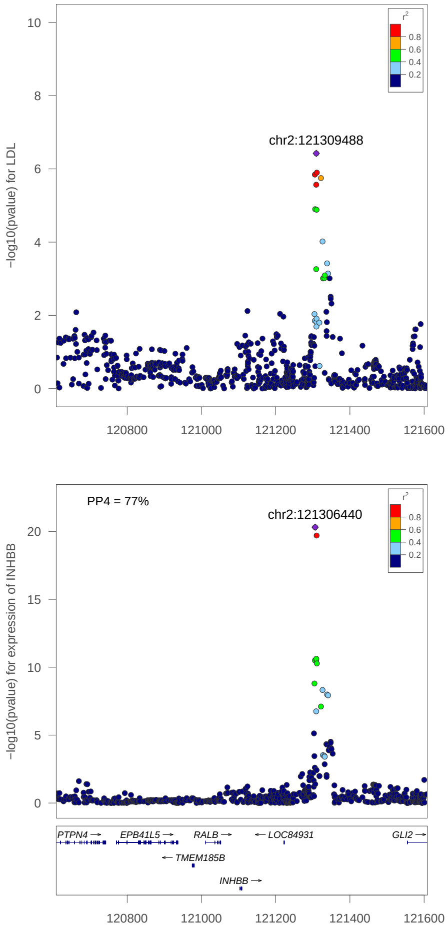

(3) INHBB/LDL

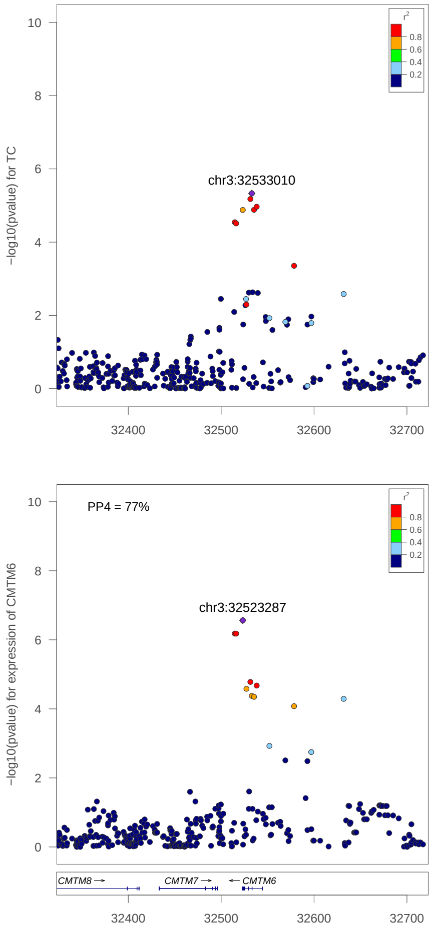

(4) CMTM6/TC

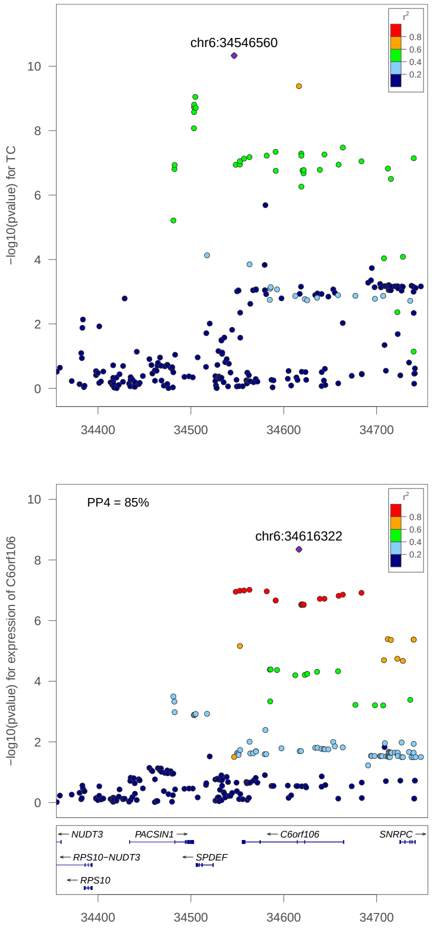

(5) C6orf106/TC

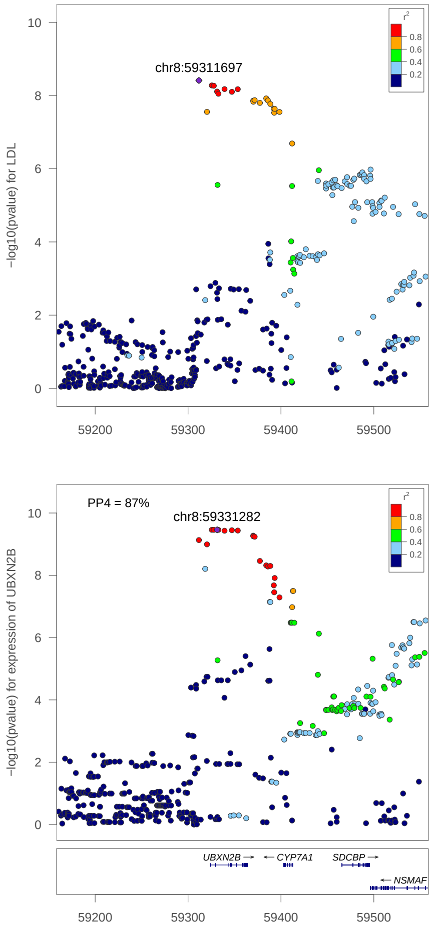

(6) UBXN2B/LDL

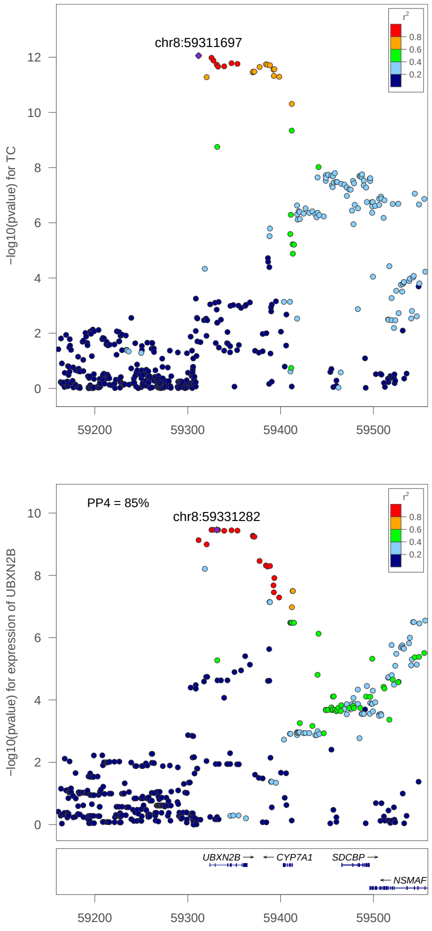

(7) UBXN2B/TC

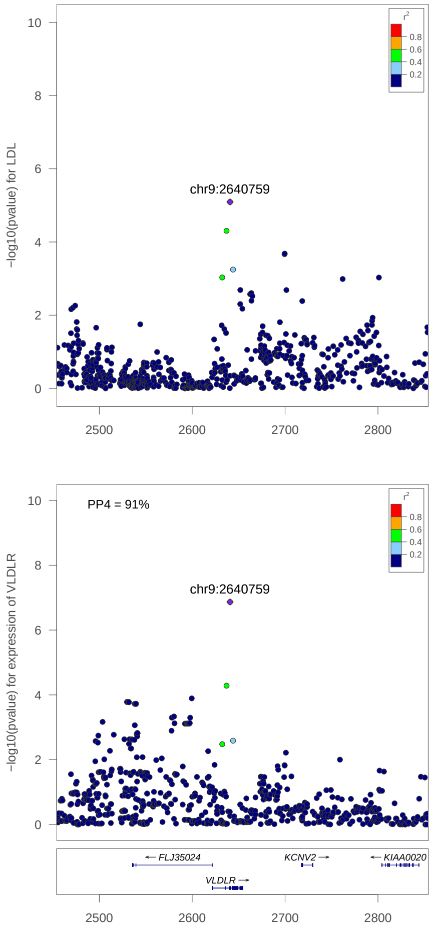

(8) VLDLR/LDL

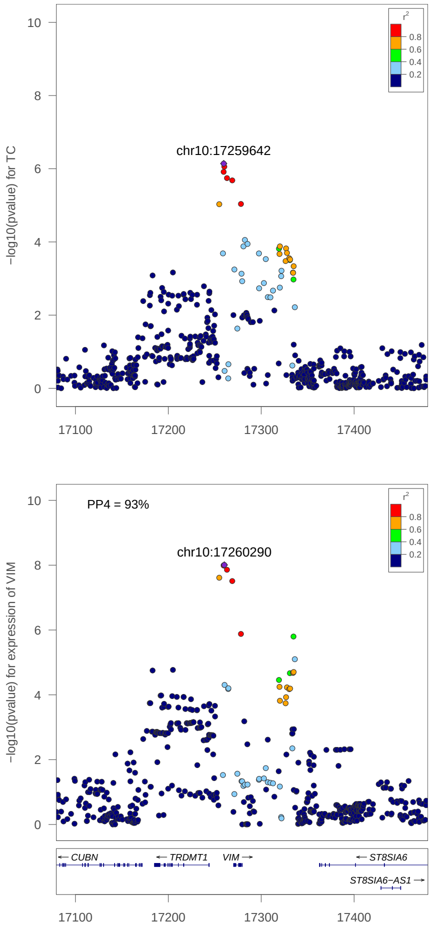

(9) VIM/TC

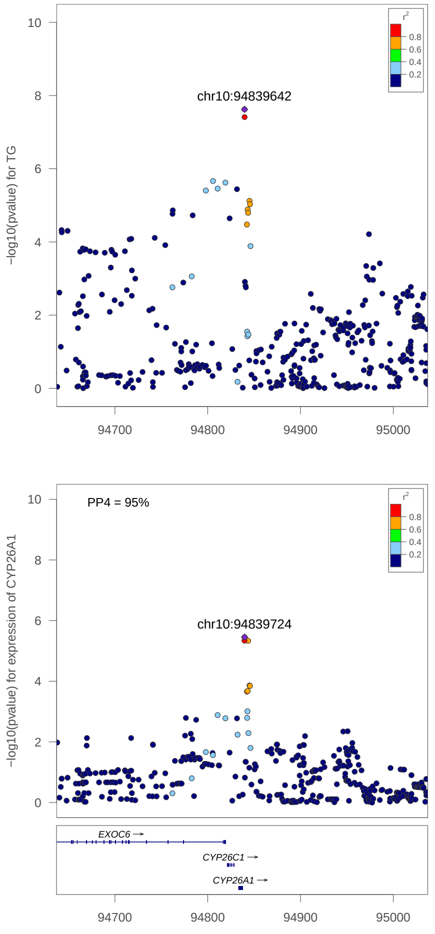

(10) CYP26A1/TG

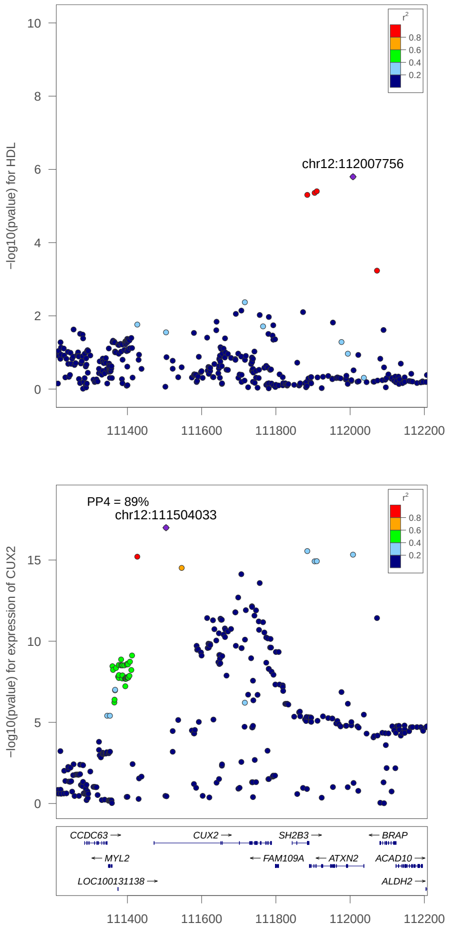

(11) CUX2/HDL

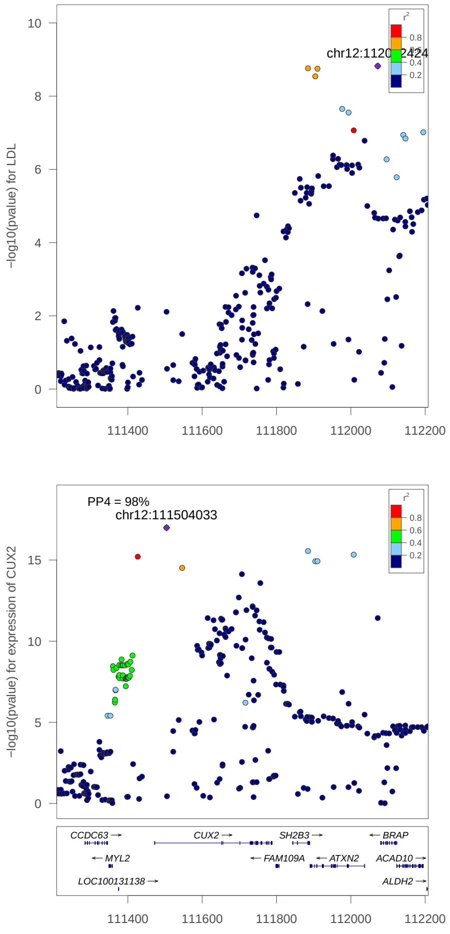

(12) CUX2/LDL

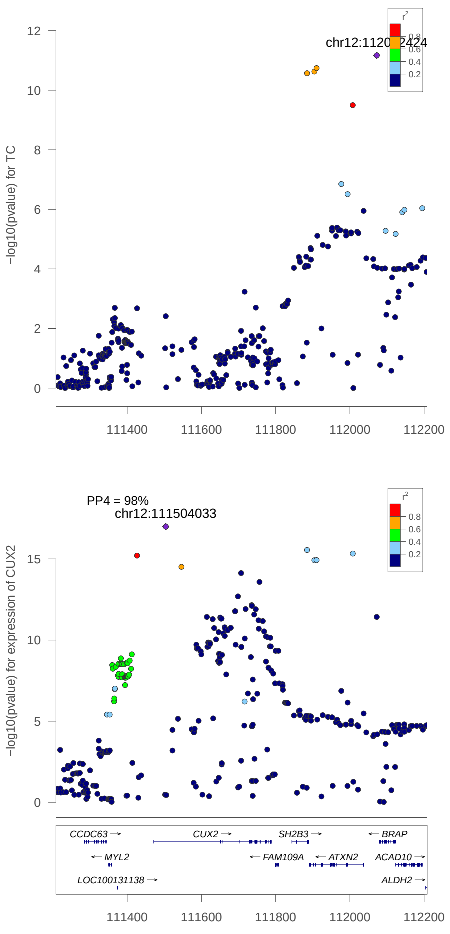

(13) CUX2/TC

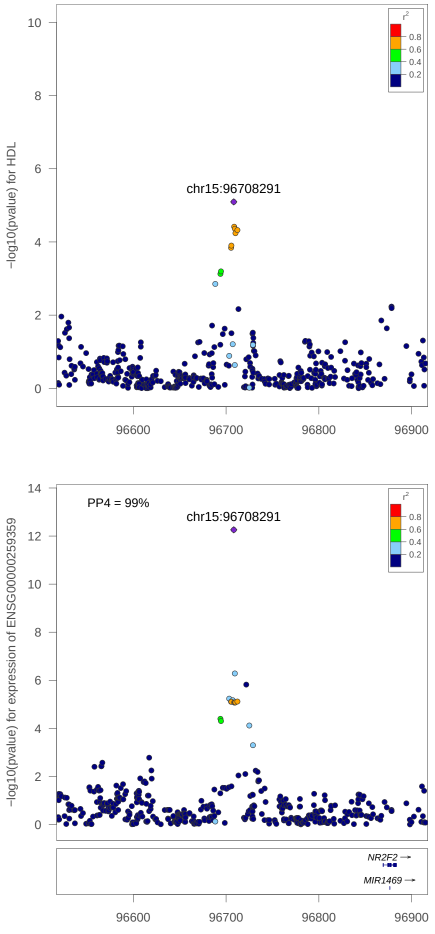

(14) ENSG00000259359/HDL

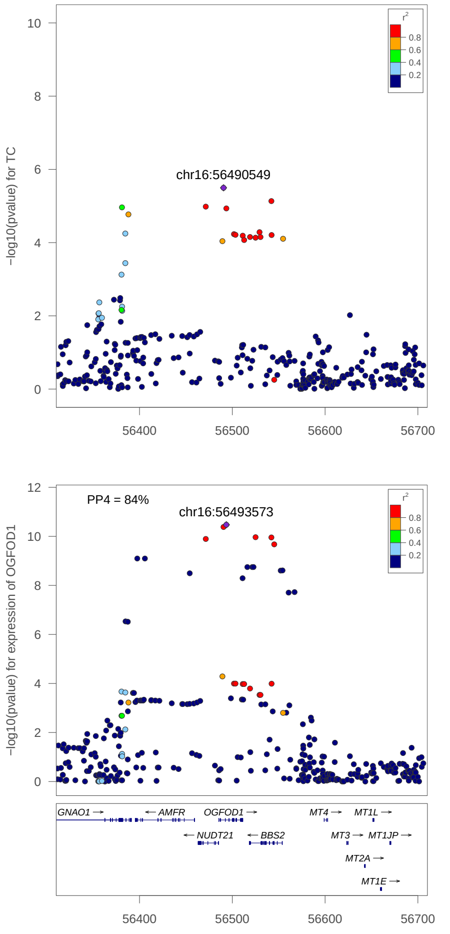

(15) OGFOD1/TC

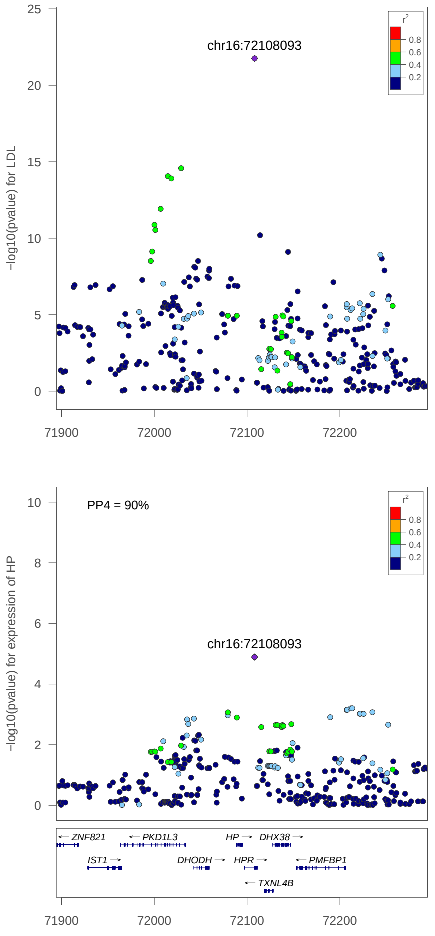

(16) HP/LDL

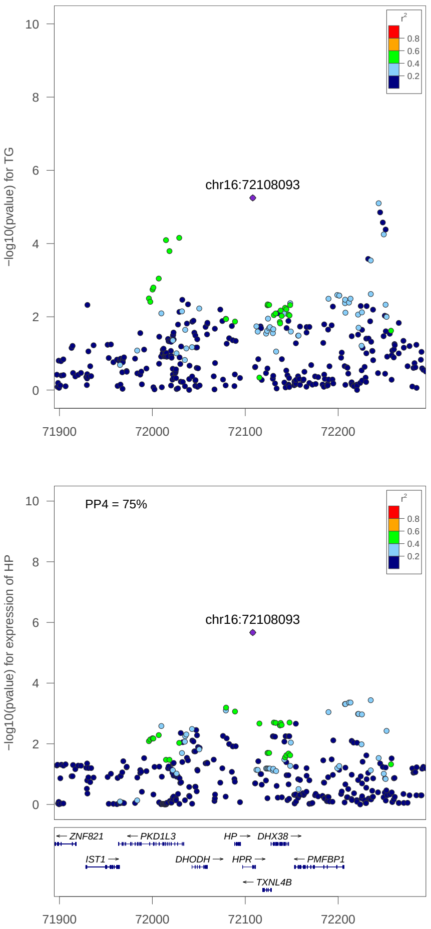

(17) HP/TG

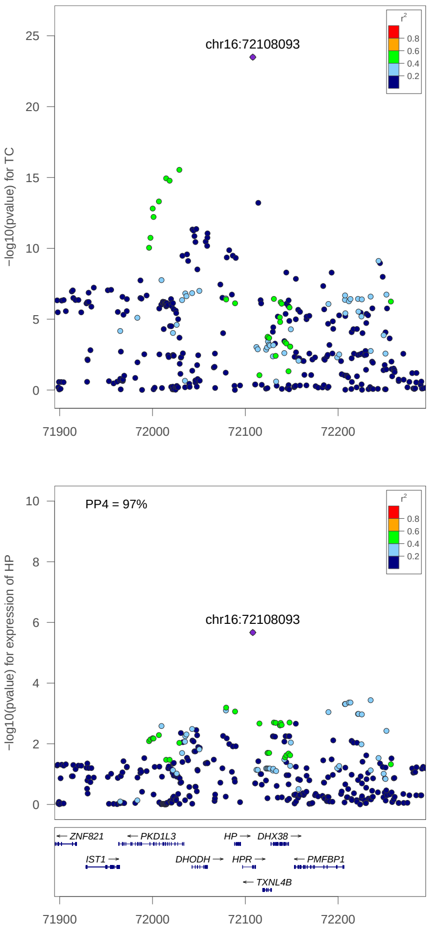

(18) HP/TC

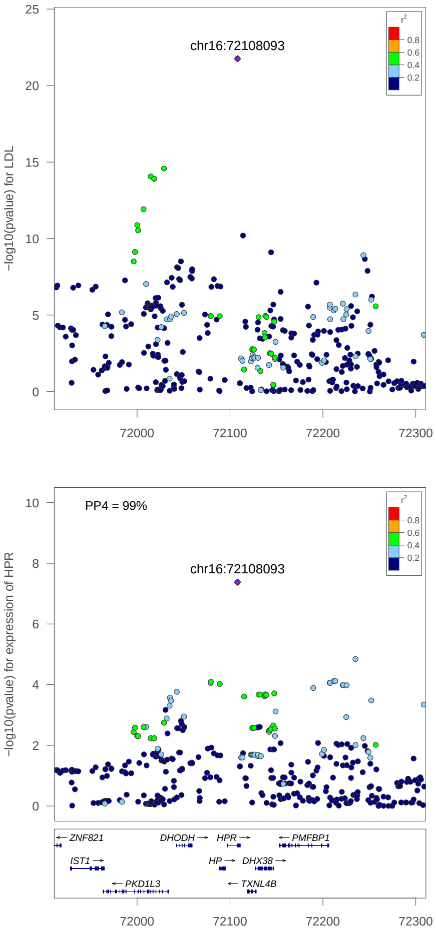

(19) HPR/LDL

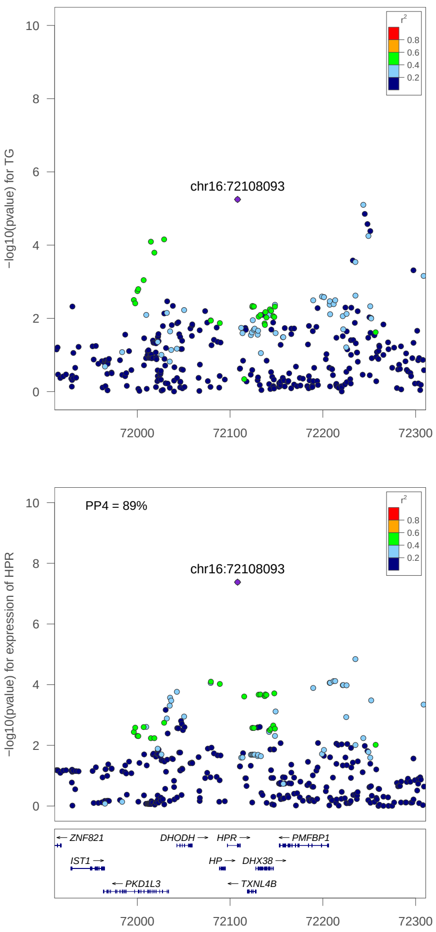

(20) HPR/TG

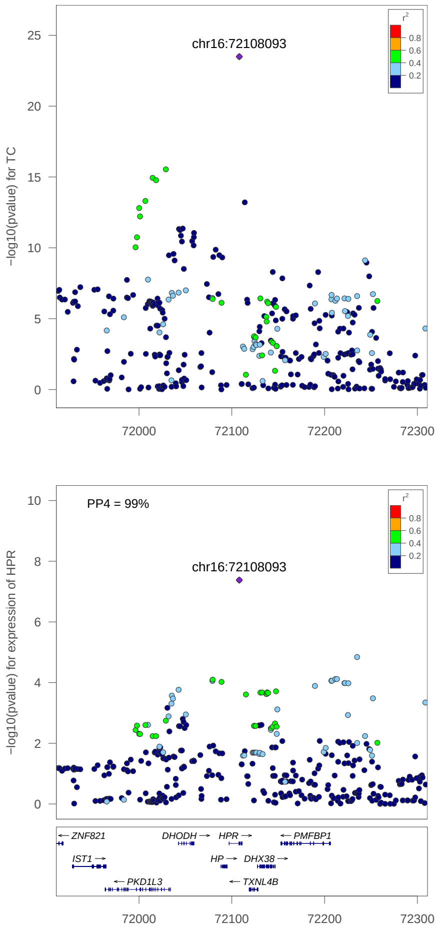

(21) HPR/TC

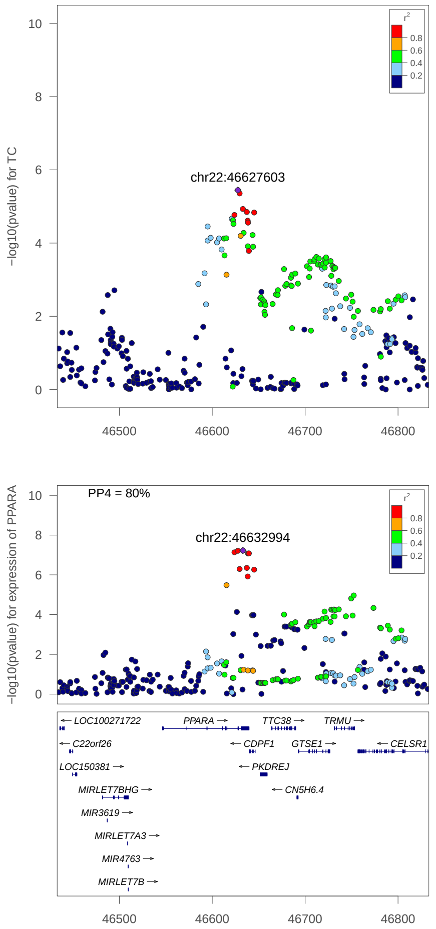

(22) PPARA/TC
