# Supplementary material for: Bayesian Test for Colocalisation between Pairs of Genetic Association Studies Using Summary Statistics
Source: PLoS Genet. 2014 May 15;10(5):e1004383. doi: 10.1371/journal.pgen.1004383 (PMC4022491; doi:10.1371/journal.pgen.1004383)
Supplement: Table S1 — Results using reported loci that colocalise with liver eQTL. Published results of loci correlating with both liver expression and one of the four lipid traits (Teslovich et al. Supplementary Table 8) and posterior probability of different signal (PP3) and common signal (PP4) after applying colocalisation test. Each row lists the results for one probe, and the multiple entries for the same locus and trait represent multiple probes mapping to the same locus. the columns Biom pval and eQTL pval report the lowest p-values found for the association with the trait listed and for the liver expression association respectively, with the corresponding SNP name (Biom SNP and eQTL SNP); the column Best Causal reports the SNP within the region with the highest posterior probability to be the true causal variant. The probabilities have been rounded to 1 significant figure. (PDF) [file pgen.1004383.s010.pdf]

Table S1. Results using reported loci that colocalise with liver eQTL

geneTranscriptsymbol	TranscriptPvalueTesl	Trait	Chr	Region	Biom pval	Biom SNP	eQTL pval	eQTL SNP	Best Causal	$p_{12} = 10^{-5}$		$p_{12} = 2 \times 10^{-6}$		$p_{12} = 10^{-6}$	
										PP3 (%)	PP4 (%)	PP3 (%)	PP4 (%)	PP3 (%)	PP4 (%)
RHCE	7.00E-54	LDL	1	25488927:25888986	1.24E-10	rs12027135	1.20E-66	rs686631	rs12027135	1	99	6	94	12	88
			1	25515852:25915911	1.24E-10	rs12027135	1.20E-16	rs909832	rs12027135	84	16	96	4	98	2
		TC	1	25488927:25888986	4.1E-11	rs12027135	7.2E-66	rs686631	rs11802413	1	99	6	94	11	89
			1	25515852:25915911	4.1E-11	rs12027135	1.2E-16	rs909832	rs3091242	85	15	97	3	98	2
RHD	4.00E-08	LDL	1	25428038:25828097	1.20E-10	rs12027135	7.70E-11	rs909832	rs12027135	9	91	33	67	50	50
			1	25456834:25856893	1.20E-10	rs12027135	0.0087	rs909832	rs12027135	< 1	2	< 1	< 1	< 1	< 1
		TC	1	25428038:25828097	4.10E-11	rs12027135	7.70E-11	rs909832	rs12027135	9	91	33	67	50	50
			1	25456834:25856893	4.10E-11	rs12027135	0.0087	rs909832	rs12027135	< 1	2	< 1	< 1	< 1	< 1
TMEM50A	4.00E-08	LDL	1	25488669:25888728	1.20E-10	rs12027135	4.40E-11	rs9689	rs3091242	12	88	41	59	58	42
		TC	1	25488669:25888728	4.10E-11	rs12027135	4.40E-11	rs9689	rs3091242	13	87	43	57	60	39
TMEM57	2.00E-145	LDL	1	25626305:26026364	1.20E-10	rs12027135	2.10E-31	rs873308	rs12027135	1	99	5	94	10	90
			1	25624780:26024839	1.20E-10	rs12027135	6.40E-08	rs686631	rs10903129	2	98	9	90	17	82
			1	25625807:26025866	1.20E-10	rs12027135	7.60E-223	rs873308	rs873308	2	97	11	88	21	79
		TC	1	25626305:26026364	4.10E-11	rs12027135	2.10E-31	rs873308	rs12027135	1	99	5	94	11	89
			1	25624780:26024839	4.10E-11	rs12027135	6.40E-08	rs686631	rs12027135	2	98	9	90	17	82
			1	25625807:26025866	4.10E-11	rs12027135	7.60E-223	rs873308	rs873308	3	97	15	85	26	74
ANGPTL3	1.00E-13	LDL	1	62870388:63270447	2.60E-18	rs3850634	1.90E-15	rs636497	rs3850634	9	91	34	66	51	49
		TC	1	62870388:63270447	4.90E-41	rs3850634	1.90E-15	rs636497	rs3850634	9	90	34	65	51	49
		TG	1	62870388:63270447	8.80E-43	rs2131925	1.90E-15	rs636497	rs2131925	8	92	29	70	45	54
DOCK7	1.00E-22	LDL	1	62849818:63249877	2.60E-18	rs3850634	1.90E-25	rs11485618	rs3850634	5	95	23	77	37	63
			1	62720869:63120928	2.60E-18	rs3850634	0.0049	rs10458569	rs3850634	1	13	2	3	2	1
		TC	1	62849818:63249877	4.90E-41	rs3850634	1.90E-25	rs11485618	rs10789118	5	95	20	80	33	67
			1	62720869:63120928	4.90E-41	rs3850634	0.0049	rs10458569	rs3850634	1	14	2	3	2	1
		TG	1	62849818:63249877	8.80E-43	rs2131925	1.90E-25	rs11485618	rs2131925	5	94	23	77	37	63
			1	62720869:63120928	8.80E-43	rs2131925	0.0049	rs10458569	rs2131925	1	14	2	3	2	2
CELSR2	5.00E-94	LDL	1	109618271:110018330	9.70E-171	rs629301	1.50E-120	rs646776	rs629301	< 1	> 99	1	99	2	98
		TC	1	109618271:110018330	5.80E-131	rs629301	1.50E-120	rs646776	rs629301	< 1	> 99	1	99	3	97
PSMA5	9.00E-17	LDL	1	109744528:110144587	9.70E-171	rs629301	1.50E-17	rs599839	rs629301	< 1	99	4	96	7	93
			1	109741904:110141963	9.70E-171	rs629301	1.20E-07	rs600806	rs629301	98	1	99	< 1	99	< 1
		TC	1	109744528:110144587	5.80E-131	rs629301	1.50E-17	rs599839	rs629301	< 1	99	3	97	7	93
			1	109741904:110141963	5.80E-131	rs629301	1.20E-07	rs600806	rs629301	98	1	99	< 1	99	< 1
PSRC1	2.00E-271	LDL	1	109622208:110022267	9.70E-171	rs629301	1.10E-299	rs7528419	rs629301	< 1	99	3	97	7	93
		TC	1	109622208:110022267	5.80E-131	rs629301	1.10E-299	rs7528419	rs629301	< 1	99	3	96	7	93
SORT1	2.00E-300	LDL	1	109652373:110052432	9.70E-171	rs629301	1.10E-299	rs7528419	rs629301	< 1	> 99	3	96	7	93
			1	109656429:110056488	9.70E-171	rs629301	1.10E-299	rs7528419	rs629301	< 1	> 99	3	96	7	93
		TC	1	109652373:110052432	5.80E-131	rs629301	1.10E-299	rs7528419	rs629301	< 1	> 99	3	96	7	93
			1	109656429:110056488	5.80E-131	rs629301	1.10E-299	rs7528419	rs629301	< 1	> 99	3	96	7	93
SYPL2	1.00E-23	LDL	1	109024678:111024737	9.70E-171	rs629301	7.10E-103	rs2359653	rs629301	> 99	< 1	> 99	< 1	> 99	< 1
			1	109821999:110222054	2.90E-168	rs599839	0.0031	rs7536292	rs599839	< 1	< 1	< 1	< 1	< 1	< 1
		TC	1	109824678:110224737	8.00E-52	rs672569	7.10E-103	rs1933182	rs1933182	> 99	< 1	> 99	< 1	> 99	< 1
			1	109821999:110222054	4.10E-130	rs599839	0.0031	rs7536292	rs599839	< 1	< 1	< 1	< 1	< 1	< 1
IFT172	7.00E-32	TC	2	27467244:27867303	7.30E-27	rs1260326	1.70E-130	rs704791	rs704791	> 99	< 1	> 99	< 1	> 99	< 1
		TG	2	27467244:27867303	5.70E-133	rs1260326	1.70E-130	rs704791	rs1260326	> 99	< 1	> 99	< 1	> 99	< 1
SLC39A8	3.00E-19	HDL	4	102982958:103383017	7.20E-11	rs13107325	3.80E-21	rs13107325	rs13107325	< 1	> 99	< 1	> 99	< 1	> 99
			4	102972446:103372505	7.20E-11	rs13107325	0.027	rs11733483	rs13107325	< 1	< 1	< 1	< 1	< 1	< 1
HLA-DQB1	2.00E-13	TC	6	32427977:32828028	2.60E-17	rs17533167	2.10E-43	rs3129720	rs3129720	> 99	< 1	> 99	< 1	> 99	< 1
HLA-DRB1	7.00E-44	TC	6	32346553:32746610	4.00E-19	rs3177928	1.90E-217	rs477515	rs477515	> 99	< 1	> 99	< 1	> 99	< 1
FRK	4.00E-12	LDL	6	116062804:116462863	2.90E-09	rs11153594	6.60E-15	rs195517	rs195517	90	10	98	2	99	1
		TC	6	116062804:116462863	1.70E-10	rs9488822	6.60E-15	rs195517	rs9488822	61	39	89	11	94	6
PPP1R3B	1.00E-14	LDL	8	8795514:9195573	7.40E-15	rs2126259	6.20E-17	rs2126259	rs2126259	< 1	> 99	2	98	4	96
			8	8793929:9193988	7.40E-15	rs2126259	1.90E-17	rs4240624	rs9987289	1	99	5	94	10	89
		TC	8	8795514:9195573	9.00E-24	rs2126259	6.20E-17	rs2126259	rs2126259	< 1	> 99	2	98	3	97
			8	8793929:9193988	9.00E-24	rs2126259	1.90E-17	rs4240624	rs2126259	1	98	8	92	14	85
		HDL	8	8793929:9193988	6.40E-25	rs9987289	6.20E-17	rs2126259	rs9987289	< 1	> 99	2	98	4	96
			8	8795514:9195573	6.40E-25	rs9987289	1.90E-17	rs4240624	rs9987289	1	98	7	93	14	86
TTC39B	2.00E-15	HDL	9	14971602:15371661	1.30E-13	rs643531	8.10E-18	rs581080	rs686030	2	98	10	89	19	81
SPTY2D1	1.00E-16	TC	11	18429356:18829415	2.50E-08	rs10832963	7.20E-17	rs10832963	rs10832963	< 1	99	3	97	6	94
			11	18427988:18828047	2.50E-08	rs10832963	2.90E-19	rs10832963	rs10832963	< 1	99	3	97	6	94
FADS1	5.00E-18	LDL	11	61367291:61767350	1.20E-21	rs174583	2.90E-20	rs102275	rs102275	2	98	9	91	17	83
		TC	11	61367291:61767350	2.10E-22	rs174550	2.90E-20	rs102275	rs102275	1	99	7	93	13	87
		TG	11	61367291:61767350	5.40E-24	rs174546	2.90E-20	rs102275	rs102275	1	99	5	95	10	90
		HDL	11	61367291:61767350	1.50E-22	rs174601	2.90E-20	rs102275	rs102275	< 1	99	4	96	8	92
ST3GAL4	2.00E-22	LDL	11	126084467:126484526	1.20E-15	rs11220462	7.20E-25	rs4307732	rs4307732	2	98	8	92	15	85
		TC	11	126084467:126484526	2.10E-11	rs11220463	7.20E-25	rs4307732	rs7951028	1	99	7	93	13	87
MMAB	2.00E-44	HDL	12	109793364:110193423	6.90E-15	rs7134594	1.00E-38	rs7954144	rs7954144	2	97	12	88	21	79
NYNRIN	3.00E-46	LDL	14	24688259:25088318	4.40E-11	rs2332328	1.10E-78	rs6573778	rs6573778	< 1	99	3	97	5	95
CKMT1A	8.00E-28	TG	15	43791310:44191369	4.70E-08	rs9989313	1.20E-13	rs2260160	rs9989313	5	95	20	79	33	66
ALDH1A2	5.00E-08	TC	15	58334099:58734158	8.80E-20	rs1532085	5.00E-45	rs1532085	rs1532085	< 1	> 99	1	99	2	98
			15	58045824:58445883	0.0042	rs4646590	0.0027	rs9796698	rs9796698	< 1	< 1	< 1	< 1	< 1	< 1
		HDL	15	58334099:58734158	2.90E-96	rs1532085	5.00E-45	rs1532085	rs1532085	< 1	> 99	< 1	> 99	1	99
			15	58045824:58445883	0.0014	rs6493966	0.0027	rs9796698	rs8040266	< 1	< 1	< 1	< 1	< 1	< 1
		TG	15	58334099:58734158	2.40E-13	rs261342	5.00E-45	rs1532085	rs2043085	4	96	18	81	31	69
			15	58045824:58445883	0.0046	rs2414536	0.0027	rs9796698	rs9796698	< 1	< 1	< 1	< 1	< 1	< 1
LIPC	7.00E-23	TC	15	58653103:59053162	8.80E-20	rs1532085	1.10E-25	rs2043085	rs2043085	< 1	> 99	1	99	3	97
		HDL	15	58653103:59053162	2.90E-96	rs1532085	1.10E-25	rs2043085	rs1532085	< 1	> 99	2	98	3	97
		TG	15	58653103:59053162	2.40E-13	rs261342	1.10E-25	rs2043085	rs2043085	2	98	10	89	19	81
VKORC1	7.00E-47	TG	16	30904631:31304682	3.30E-08	rs11649653	1.10E-80	rs749671	rs749671	3	97	13	87	22	77
NFATC3	3.00E-15	HDL	16	68060252:68460297	1.20E-27	rs3785108	3.30E-05	rs7204208	rs3785108	3	91	10	68	15	51
			16	68051875:68451934	1.20E-27	rs3785108	0.0067	rs3743739	rs3785108	< 1	5	< 1	< 1	< 1	< 1
			16	68025772:68425831	1.20E-27	rs3785108	0.00055	rs9930867	rs3785108	2	< 1	2	< 1	2	< 1
			16	68063016:68463075	1.20E-27	rs3785108	0.0068	rs1868158	rs3785108	< 1	< 1	< 1	< 1	< 1	< 1
			16	68060703:68460762	1.20E-27	rs3785108	4.20E-30	rs7193701	rs7204208	> 99	< 1	> 99	< 1	> 99	< 1
PERLD1/PGAP3	9.00E-24	HDL	17	37627395:38027454	2.84E-14	rs881844	6.51E-33	rs2941506	rs2941505	14	86	45	54	63	37

TBKBP1	6.00E-10	TC	17	45589357:45989416	1.80E-07	rs8072100	2.10E-21	rs9913503	rs4794053	78	17	90	4	92	2
		LDL	17	45589357:45989416	1.10E-07	rs8072100	2.10E-21	rs9913503	rs6503807	47	51	79	17	87	9
LIPG	4.00E-10	TC	18	46918514:47318573	2.00E-19	rs7239867	1.20E-11	rs4939883	rs4939883	1	99	5	94	11	89
		HDL	18	46918514:47318573	2.70E-49	rs7241918	1.20E-11	rs4939883	rs4939883	< 1	> 99	2	98	4	96
ANGPTL4	4.00E-08	HDL	19	8239194:8639253	3.20E-08	rs7255436	3.80E-09	rs7255436	rs7255436	< 1	> 99	2	98	5	95
APOC4	4.00E-09	TG	19	45248464:45648523	1.10E-30	rs439401	9.40E-15	19:45430280	rs439401	98	2	> 99	< 1	> 99	< 1
			19	45252653:45652712	1.10E-30	rs439401	1.10E-299	rs1130742	rs1130742	> 99	< 1	> 99	< 1	> 99	< 1
LILRA3	9.00E-12	HDL	19	54602026:55002085	4.30E-16	rs386000	8.20E-17	19:54793830	rs386000	< 1	> 99	4	95	9	91
CEP250	3.00E-08	TC	20	33899702:34299761	3.80E-10	rs2277862	0.022	rs17424259	rs2104417	< 1	< 1	< 1	< 1	< 1	< 1
CPNE1	7.00E-41	TC	20	34013995:34414054	3.80E-10	rs2277862	7.30E-110	rs6060524	rs6060524	95	5	99	1	99	< 1
PLTP	3.00E-18	TG	20	44327404:44727463	4.70E-18	rs4810479	1.80E-20	rs6065906	rs4810479	< 1	> 99	3	97	5	95
		HDL	20	44327404:44727463	1.90E-22	rs6065906	1.80E-20	rs6065906	rs6065906	< 1	> 99	2	98	5	95
UBE2L3	6.00E-13	HDL	22	21778264:22178323	1.10E-08	22:21932068	8.90E-13	rs4821112	rs2283790	3	96	16	84	27	72

Published results of loci correlating with both liver expression and one of the four lipid traits (Teslovich et al. Supplementary Table 8) and posterior probability of different signal (PP3) and common signal (PP4) after applying colocalisation test. Each row lists the results for one probe, and the multiple entries for the same locus and trait represent multiple probes mapping to the same locus. the columns **Biom pval** and **eQTL pval** report the lowest p-value found for the association with the trait listed and for the liver expression association respectively (after the datasets have been combined), with the corresponding SNP name (**Biom SNP** and **eQTL SNP**); the column **Best Causal** reports the SNP within the region with the highest posterior probability to be the true causal variant. The probabilities have been rounded to 1 significant figure.
