# Supplementary material for: Bayesian Test for Colocalisation between Pairs of Genetic Association Studies Using Summary Statistics
Source: PLoS Genet. 2014 May 15;10(5):e1004383. doi: 10.1371/journal.pgen.1004383 (PMC4022491; doi:10.1371/journal.pgen.1004383)
Supplement: Table S2 — eQTL/LDL colocalisation. Positive (PP4 >75%) eQTL/LDL colocalisation results between the liver eQTL dataset and the Teslovich meta-analysis using the most stringent prior for the probability that one SNP is associated with both traits, . The column Signal includes genes that are part of overlapping regions and that colocalise at PP4 >75%; the column Region represents the genomic coordinates for the start and stop of the signal; in the column Tesl, “Y” indicates that this signal with any of the genes included has been reported to be an intermediate for any of the four lipid biomarker associations by Teslovich et al. ; the columns Biom pval and eQTL pval report the lowest p-values found for LDL association and for the expression association respectively, with the corresponding SNP name (Biom SNP and eQTL SNP); the column Best Causal reports the SNP within the region with the highest posterior probability to be the true causal variant. The probabilities have been rounded to 1 significant figure. (PDF) [file pgen.1004383.s011.pdf]

Table S2. eQTL/LDL colocalisation

Chr	Region	Signal	PP.H3.abf	PP.H4.abf	Tesl	Biom pval	Biom SNP	eQTL pval	eQTL SNP	Best Causal
1	109618271:110144587	CELSR2	2	98	Y	9.70E-171	rs629301	1.50E-120	rs646776	rs629301
		PSRC1	7	93	Y	9.70E-171	rs629301	1.10E-299	rs7528419	rs629301
		SORT1	7	93	Y	9.70E-171	rs629301	1.10E-299	rs7528419	rs629301
		PSMA5	7	93	Y	9.70E-171	rs629301	1.50E-17	rs599839	rs629301
1	150767063:151167122	ANXA9	3	97	N	6.50E-08	rs267733	6.10E-08	rs267734	rs267733
1	25626305:26026364	TMEM57	10	90	Y	1.20E-10	rs12027135	2.10E-31	rs873308	rs12027135
2	120908798:121308857	INHBB	7	77	N	3.80E-07	rs2030746	4.90E-21	rs17050272	rs17050272
8	59158506:59558565	UBXN2B	13	87	N	3.90E-09	rs1030431	3.50E-10	rs11996829	rs13263105
8	8795514:9392282	PPP1R3B	4	96	Y	7.40E-15	rs2126259	6.20E-17	rs2126259	rs2126259
		ENSG00000254235	13	87	N	7.40E-15	rs2126259	4.00E-13	rs4841133	rs9987289
9	2454062:2854121	VLDLR	1	91	N	8.00E-06	rs3780181	1.40E-07	rs3780181	rs3780181
11	126084467:126484526	ST3GAL4	15	85	Y	1.20E-15	rs11220462	7.20E-25	rs4307732	rs4307732
11	18429356:18829415	SPTY2D1	6	93	N	2.90E-07	rs10128711	7.20E-17	rs10832963	rs10832963
11	61367291:61767350	FADS1	17	83	Y	1.20E-21	rs174583	2.90E-20	rs102275	rs102275
12	111508189:111908248	CUX2	2	98	N	1.50E-09	rs11065987	1.00E-17	rs4378452	rs3184504
14	24688259:25088318	NYNRIN	5	95	Y	4.40E-11	rs2332328	1.10E-78	rs6573778	rs6573778
16	71894416:72310900	HP	1	97	N	1.80E-22	rs2000999	1.30E-05	rs2000999	rs2000999
		HPR	1	99	N	1.80E-22	rs2000999	4.20E-08	rs2000999	rs2000999
17	45562645:45962704	KPNB1	16	83	N	1.10E-07	rs8072100	3.10E-09	rs4794048	rs8072100
20	12947054:13347113	SPTLC3	4	86	N	4.20E-06	rs364585	2.30E-41	rs168622	rs364585

Positive (PP4 > 75%) eQTL/LDL colocalisation results between the liver eQTL dataset and the Teslovich meta-analysis using the most stringent prior for the probability that one SNP is associated with both traits, $p_{12} = 10^{-6}$. The column **Signal** includes genes that are part of overlapping regions and that colocalise at PP4 > 75%; the column **Region** represents the genomic coordinates for the start and stop of the signal; in the column **Tesl**, “Y” indicates that this signal with any of the genes included has been reported to be an intermediate for any of the four lipid biomarker associations by Teslovich et al. ; the columns **Biom pval** and **eQTL pval** report the lowest p-value found for LDL association and for the expression association respectively, with the corresponding SNP name (**Biom SNP** and **eQTL SNP**); the column **Best Causal** reports the SNP within the region with the highest posterior probability to be the true causal variant. The probabilities have been rounded to 1 significant figure.
