# Supplementary material for: Bayesian Test for Colocalisation between Pairs of Genetic Association Studies Using Summary Statistics
Source: PLoS Genet. 2014 May 15;10(5):e1004383. doi: 10.1371/journal.pgen.1004383 (PMC4022491; doi:10.1371/journal.pgen.1004383)
Supplement: Table S3 — eQTL/HDL colocalisation. Positive (PP4 >75%) eQTL/HDL colocalisation results between the liver eQTL dataset and the Teslovich meta-analysis. Column and row headings are the same as in previous figure. (PDF) [file pgen.1004383.s012.pdf]

Table S3. eQTL/HDL colocalisation

Chr	Region	Signal	PP.H3.abf	PP.H4.abf	Tesl	Biom pval	Biom SNP	eQTL pval	eQTL SNP	Best Causal
1	109618271:110144587	CELSR2	3	97	N	6.20E-08	rs629301	1.50E-120	rs646776	rs646776
		PSRC1	7	93	N	6.20E-08	rs629301	1.10E-299	rs7528419	rs629301
		SORT1	7	93	N	6.20E-08	rs629301	1.10E-299	rs7528419	rs629301
		PSMA5	7	92	N	6.20E-08	rs629301	1.50E-17	rs599839	rs12740374
2	85349026:85749085	TGOLN2	17	83	N	1.00E-07	rs1053560	2.80E-80	rs1044973	rs1044973
4	102982958:103383017	SLC39A8	1	99	Y	7.20E-11	rs13107325	3.80E-21	rs13107325	rs13107325
8	8795514:9392282	PPP1R3B	14	86	Y	6.40E-25	rs9987289	1.90E-17	rs4240624	rs9987289
		ENSG00000254235	4	96	N	6.40E-25	rs9987289	4.00E-13	rs4841133	rs9987289
9	14971602:15371661	TTC39B	19	81	Y	1.30E-13	rs643531	8.10E-18	rs581080	rs686030
11	61367291:61767350	FADS1	8	92	Y	1.50E-22	rs174601	2.90E-20	rs102275	rs102275
12	109793364:110193423	MMAB	21	79	Y	6.90E-15	rs7134594	1.00E-38	rs7954144	rs7954144
12	111508189:111908248	CUX2	2	89	N	4.40E-06	rs4766578	2.80E-16	rs3184504	rs3184504
15	58334099:59053162	ALDH1A2	1	99	Y	2.90E-96	rs1532085	5.00E-45	rs1532085	rs1532085
		LIPC	3	97	Y	2.90E-96	rs1532085	1.10E-25	rs2043085	rs1532085
15	96517293:96917352	ENSG00000259359	2	87	N	8.00E-06	rs8023580	5.50E-13	rs8023580	rs8023580
18	46918514:47318573	LIPG	4	96	Y	2.70E-49	rs7241918	1.20E-11	rs4939883	rs4939883
19	54578320:55002085	LILRB2	9	88	N	4.30E-16	rs386000	1.70E-06	rs416867	rs386000
		LILRA3	9	91	Y	4.30E-16	rs386000	8.20E-17	19:54793830	rs386000
19	8239194:8639253	ANGPTL4	5	95	Y	3.20E-08	rs7255436	3.80E-09	rs7255436	rs7255436
20	44327404:44727463	PLTP	5	95	Y	1.90E-22	rs6065906	1.80E-20	rs6065906	rs6065906

Positive (PP4 > 75%) eQTL/HDL colocalisation results between the liver eQTL dataset and the Teslovich meta-analysis. Column and row headings are the same as in previous figure.
