# Supplementary material for: Bayesian Test for Colocalisation between Pairs of Genetic Association Studies Using Summary Statistics
Source: PLoS Genet. 2014 May 15;10(5):e1004383. doi: 10.1371/journal.pgen.1004383 (PMC4022491; doi:10.1371/journal.pgen.1004383)
Supplement: Table S4 — eQTL/TG colocalisation. Positive (PP4 >75%) eQTL/HDL colocalisation results between the liver eQTL dataset and the Teslovich meta-analysis. Column and row headings are the same as in previous figure. (PDF) [file pgen.1004383.s013.pdf]

Table S4. eQTL/TG colocalisation

Chr	Region	Signal	PP.H3.abf	PP.H4.abf	Tesl	Biom pval	Biom SNP	eQTL pval	eQTL SNP	Best Causal
2	2754647:28005583	GCKR	5	77	N	5.70E-133	rs1260326	1.50E-05	rs1260326	rs1260326
		C2orf16	4	81	N	5.70E-133	rs1260326	8.30E-06	rs1260326	rs1260326
10	94637063:95037122	CYP26A1	3	95	N	2.40E-08	rs2068888	3.50E-06	rs4418728	rs2068888
11	61367291:61767350	FADS1	10	90	Y	5.40E-24	rs174546	2.90E-20	rs102275	rs102275
15	58653103:59053162	LIPC	19	81	Y	2.40E-13	rs261342	1.10E-25	rs2043085	rs2043085
16	30904631:31304682	VKORC1	23	77	Y	3.30E-08	rs11649653	1.10E-80	rs749671	rs749671
		ENSG00000255439	23	77	N	3.30E-08	rs11649653	1.10E-80	rs749671	rs749671
16	71894416:72310900	HP	2	75	N	5.70E-06	rs2000999	2.10E-06	rs2000999	rs2000999
		HPR	2	89	N	5.70E-06	rs2000999	4.20E-08	rs2000999	rs2000999
20	44327404:44727463	PLTP	5	95	Y	4.70E-18	rs4810479	1.80E-20	rs6065906	rs4810479

Positive (PP4 $>$ 75%) eQTL/TG colocalisation results between the liver eQTL dataset and the Teslovich meta-analysis. Column and row headings are the same as in previous figure.
