# Supplementary material for: Bayesian Test for Colocalisation between Pairs of Genetic Association Studies Using Summary Statistics
Source: PLoS Genet. 2014 May 15;10(5):e1004383. doi: 10.1371/journal.pgen.1004383 (PMC4022491; doi:10.1371/journal.pgen.1004383)
Supplement: Table S5 — eQTL/TC colocalisation. Positive (PP4 >75%) eQTL/HDL colocalisation results between the liver eQTL dataset and the Teslovich meta-analysis. Column and row headings are the same as in previous figure. (PDF) [file pgen.1004383.s014.pdf]

Table S5. eQTL/TC colocalisation

Chr	Region	Signal	PP.H3.abf	PP.H4.abf	Tesl	Biom pval	Biom SNP	eQTL pval	eQTL SNP	Best Causal
1	109618271:110144587	CELSR2	3	97	Y	5.80E-131	rs629301	1.50E-120	rs646776	rs629301
		PSRC1	7	93	Y	5.80E-131	rs629301	1.10E-299	rs7528419	rs629301
		SORT1	7	93	Y	5.80E-131	rs629301	1.10E-299	rs7528419	rs629301
		PSMA5	7	93	Y	5.80E-131	rs629301	1.50E-17	rs599839	rs629301
1	25626305:26026364	TMEM57	11	89	Y	4.10E-11	rs12027135	2.10E-31	rs873308	rs12027135
2	20201795:20601854	SDC1	17	82	N	1.20E-07	rs1473886	6.70E-09	rs907866	rs1107851
2	27546474:28005583	GCKR	5	77	N	7.30E-27	rs1260326	1.50E-05	rs1260326	rs1260326
		C2orf16	4	81	N	7.30E-27	rs1260326	8.30E-06	rs1260326	rs1260326
3	32322873:32722932	CMTM6	8	77	N	9.10E-07	rs7640978	2.70E-07	rs17029597	rs17029597
6	34355095:34755154	C6orf106	15	85	N	4.70E-11	rs2814982	4.50E-09	rs3800461	rs3800461
8	59158506:59558565	UBXN2B	15	85	N	8.80E-13	rs1030431	3.50E-10	rs11996829	rs13263105
8	8795514:9195573	PPP1R3B	3	97	Y	9.00E-24	rs2126259	6.20E-17	rs2126259	rs2126259
9	14971602:15371661	TTC39B	1	99	N	3.10E-09	rs581080	8.10E-18	rs581080	rs581080
10	17079389:17479448	VIM	5	93	N	7.20E-07	rs7903259	9.80E-09	rs10904908	rs7903259
11	126084467:126484526	ST3GAL4	13	87	Y	2.10E-11	rs11220463	7.20E-25	rs4307732	rs7951028
11	18429356:18829415	SPTY2D1	6	94	Y	2.50E-08	rs10832963	7.20E-17	rs10832963	rs10832963
11	61367291:61767350	FADS1	13	87	Y	2.10E-22	rs174550	2.90E-20	rs102275	rs102275
12	111508189:111908248	CUX2	2	98	N	2.40E-11	rs4766578	2.80E-16	rs3184504	rs3184504
14	24688259:25088318	NYNRIN	3	97	N	1.10E-07	rs6573778	1.10E-78	rs6573778	rs6573778
15	58334099:59053162	ALDH1A2	2	98	Y	8.80E-20	rs1532085	5.00E-45	rs1532085	rs1532085
		LIPC	3	97	Y	8.80E-20	rs1532085	1.10E-25	rs2043085	rs2043085
16	56310220:56710279	OGFOD1	7	84		3.20E-06	rs11644679	3.40E-11	rs11649379	rs11644679
16	71894416:72310900	HP	1	97	N	3.20E-24	rs2000999	2.10E-06	rs2000999	rs2000999
		HPR	1	99	N	3.20E-24	rs2000999	4.20E-08	rs2000999	rs2000999
17	45562645:45962704	KPNB1	13	86	N	1.80E-07	rs8072100	3.10E-09	rs4794048	rs8072100
18	46918514:47318573	LIPG	11	89	Y	2.00E-19	rs7239867	1.20E-11	rs4939883	rs4939883
22	46433083:46833138	PPARA	10	81	N	3.60E-06	rs4253772	6.00E-08	rs11704979	rs4253772

Positive (PP4 > 75%) eQTL/TC colocalisation results between the liver eQTL dataset and the Teslovich meta-analysis. TColumn and row headings are the same as in previous figure.
