# Supplementary material for: Bayesian Test for Colocalisation between Pairs of Genetic Association Studies Using Summary Statistics
Source: PLoS Genet. 2014 May 15;10(5):e1004383. doi: 10.1371/journal.pgen.1004383 (PMC4022491; doi:10.1371/journal.pgen.1004383)
Supplement: Text S1 — Supplementary materials. Expanded methods, derivations and analyses. (PDF) [file pgen.1004383.s015.pdf]

Simplified model under assumption that all SNPs have the same prior

The probability space for a single SNP can be fully partitioned into (p_0, p_1, p_2, p_{12}), where p_0 is the prior probability that a SNP is not associated with either trait, p_1 is defined as the prior probability that the SNP is only associated with trait 1, p_2 the prior probability that the SNP is associated only with trait 2, while p_{12} is the prior probability that the SNP is associated with both traits. We therefore have:

$$p_0 + p_1 + p_2 + p_{12} = 1$$

The priors p_0, p_1, p_2, p_{12} can vary across SNPs, for example depending on minor allele frequencies, a measure of imputation quality, proximity to the promoter, function of the SNP. If however the priors do not vary across SNPs, then equation 1 in the main text becomes:

$$P(H_h \mid D) \propto \sum_{S \in S_h} P(D \mid S) P(S) \propto P(S \mid S \in S_h) \times \sum_{S \in S_h} P(D \mid S) \tag{1}$$

The proportionality (rather than equality) in this equation is a consequence of the normalisation term $P(D)$ that comes up when the causality is reversed between $P(S \mid D)$ and $P(D \mid S)$. This term is present systematically and cancels out when computing ratios. On the right side of this equation, the simplification is possible because each binary vector that belongs to the same set S_h has the same prior probability.

The prior probability of any one configuration in the different sets is:

- If $S \in S_0$, then $P(S) = p_0^Q$
- If $S \in S_1$, then $P(S) = p_0^{Q-1} \times p_1$
- If $S \in S_2$, then $P(S) = p_0^{Q-1} \times p_2$
- If $S \in S_3$, then $P(S) = p_0^{Q-2} \times p_1 \times p_2$
- If $S \in S_4$, then $P(S) = p_0^{Q-1} \times p_{12}$

Because we condition our analysis on having at most one association per trait, these probabilities should be normalised: $P(S) = p_0^Q / C$, where C is the sum of the probabilities associated with all the assignments that contain at most one association per trait. However, because subsequent derivations only consider the ratio of probabilities, the C term cancels out and this normalisation becomes unnecessary. Dividing each of these probabilities by $P(S_0)$ to obtain the ratio of prior odds, and since $p_0 \approx 1$, the second terms inside the sum in equation 3 of the main text become:

- If $S \in S_0$, then $\frac{P(S)}{P(S_0)} = \frac{p_0^Q}{p_0^Q} = 1$
- If $S \in S_1$, then $\frac{P(S)}{P(S_0)} = \frac{p_0^{Q-1}}{p_0^Q} \times p_1 = \frac{p_1}{p_0} \approx p_1$
- If $S \in S_2$, then $\frac{P(S)}{P(S_0)} = \frac{p_0^{Q-1}}{p_0^Q} \times p_2 = \frac{p_2}{p_0} \approx p_2$
- If $S \in S_3$, then $\frac{P(S)}{P(S_0)} = \frac{p_0^{Q-2}}{p_0^Q} \times p_1 \times p_2 = \frac{p_1}{p_0} \times \frac{p_2}{p_0} \approx p_1 \times p_2$
- If $S \in S_4$, then $\frac{P(S)}{P(S_0)} = \frac{p_0^{Q-1}}{p_0^Q} \times p_{12} = \frac{p_{12}}{p_0} \approx p_{12}$

To compute the first terms inside the sum in equation 3 of the main text, the BFs for each configuration in a set, we make use of the ABF derived for each SNP-trait association (section below). Two key assumptions are necessary for the following computations. Firstly that the traits are measured in unrelated individuals, and secondly that the effect sizes for the two traits are independent.

Putting the two terms together, we have:

- $\frac{P(H_0|D)}{P(H_0|D)} = 1$
- $\frac{P(H_1|D)}{P(H_0|D)} = p_1 \times \sum_{j=1}^{Q} ABF_j^1$
- $\frac{P(H_2|D)}{P(H_0|D)} = p_2 \times \sum_{j=1}^{Q} ABF_j^2$
- $\frac{P(H_3|D)}{P(H_0|D)} = p_1 \times p_2 \times \sum_{j,k,j \neq k} ABF_j^1 ABF_k^2$
- $\frac{P(H_4|D)}{P(H_0|D)} = p_{12} \times \sum_{j=1}^{Q} ABF_j^1 \times ABF_j^2$

Of note, we can also write:

$$\frac{P(H_3 \mid D)}{P(H_0 \mid D)} = p_1 \times p_2 \times \sum_{j=1}^{Q} ABF_j^1 \sum_{j=1}^{Q} ABF_j^2 - \left[\frac{p_1 \times p_2}{p_{12}} \times \frac{P(H_4 \mid D)}{P(H_0 \mid D)} \right]$$

Bayes factor computation

We assume that summary statistics for each SNP in the two datasets were obtained by fitting a generalised linear model with the phenotype as dependent variable and SNP genotype call as independent variable:

$$Y = \mu + \beta X$$

The Bayes factor quantities are estimated from summary statistics using the Asymptotic Bayes Factor derivation [20]. Using Wakefield's notations, under the null we assume that the effect size $\beta = 0$. Under the alternative, β is normally distributed with mean 0 and variance W.

To derive the ABF computation, Wakefield uses the fact that asymptotically $\hat{\beta} \rightarrow N(\beta, V)$. The distribution of the estimated regression parameters $\hat{\mu}$ and $\hat{\beta}$ is:

$$\begin{bmatrix} \hat{\mu} \\ \hat{\beta} \end{bmatrix} \sim N_{p+1} \left(\begin{bmatrix} \mu \\ \beta \end{bmatrix} \begin{bmatrix} \mathbb{I}_{\mu\mu} & \mathbb{I}_{\mu\beta} \\ \mathbb{I}_{\mu\beta}^T & \mathbb{I}_{\beta\beta} \end{bmatrix}^{-1} \right) \tag{2}$$

The intercept μ is a nuisance parameter which we can remove using the transformation:

$$\gamma = \mu + \frac{\mathbb{I}_{\mu\beta}}{\mathbb{I}_{\mu\mu}} \beta$$

In which case the previous equality becomes:

$$\begin{bmatrix} \hat{\gamma} \\ \hat{\beta} \end{bmatrix} \sim N_{p+1} \left(\begin{bmatrix} \gamma \\ \beta \end{bmatrix} \begin{bmatrix} \mathbb{I}^{*}{}_{\mu\mu} & 0 \\ 0^T & \mathbb{I}_{\beta\beta} \end{bmatrix}^{-1} \right) \tag{3}$$

This independence property can be combined with independent priors for both parameters so that one can only consider β and ignore the effect of the μ term. Hence, after applying the reparameterisation we have:

$$BF = \int \frac{f(\beta)}{f(0)} \pi(\beta) d\beta$$

Which then becomes (assuming normality and asymptotic behaviour):

$$ABF = \sqrt{1-r} \times exp\left[\frac{Z^2}{2} \times r\right]$$

where $Z = \hat{\beta}/\sqrt{V}$ is the Wald test statistic. The shrinkage factor r is the ratio of the variance of the prior and total variance ($r = W/(V+W)$). This ratio takes a value between 0 and 1 and measure the relative contributions of the prior (W) and likelihood (V) to the inference. Values of r closer to 1 indicate a larger contribution from the likelihood (i.e. the data). The asymptotic posterior distribution of β is $N(r\hat{\beta}, rV)$. As the sample size increases, V $\rightarrow$ 0 and r $\rightarrow$ 1, so that the posterior concentrates around the MLE. We can compute the Z statistic from the p-values of a standard regression output using $|z| = \Phi^{-1}(1-p/2)$, where Φ^{-1} is the inverse normal cumulative. Otherwise we can compute the Wald statistics directly if the estimated regression coefficients $\hat{\beta}$ and their variances $var(\hat{\beta})$ are supplied.

Variance of the estimated effect size $Var(\hat{\beta}) = V$

The variance of the maximum likelihood estimate $\hat{\beta}$ (denoted by V) can be approximated using the allele frequency of the variant f, the sample size N and the case control ratio s for binary outcome. It is well established that the score statistic to test whether the effect size $\beta = 0$ is:

$$U = \sum_i (Y_i - \bar{Y}) X_j$$

Score test theory asserts that the variance of U under the null is the inverse of the variance of the estimated effect size $\hat{\beta}$, also under the null. $Var(U)$ under the null can be estimated in several ways, including the standard derivation of the Fisher information matrix [52].

We assume that the genotypes X are under Hardy Weinberg equilibrium. Hence X is drawn from a binomial distribution with success parameter f, which we use to denote the allele frequency.

$$Var[X_j] = 2f_j(1-f_j)$$

where f_j is the population allele frequency of the SNP j.

When the dataset is a case-control (the trait Y is binomial):

$$Var[Y] = s(1-s)$$

where s is the proportion of cases in the population. If the outcome variable Y is continuous we assume that Y is normalised such that $Var(Y) = 1$.

Putting together these equations, we have, in a case control setting:

$$V = Var(\hat{\beta}_j) = \frac{1}{Ns(1-s) \times 2f_j(1-f_j)}$$

Choice of priors for the probability that each variant affects the traits

The prior probabilities assigned to each SNP, p_0, p_1, p_2, p_{12}, are mutually exclusive events. The probability of a SNP being associated with both traits, p_{12}, can be interpreted using a conditional argument:

$$\begin{aligned} p_{12} &= \mathbb{P}(\text{SNP associated with both traits}) \\ &= \mathbb{P}(\text{SNP associated with trait 1}) \times \mathbb{P}(\text{SNP associated with trait 2} \quad | \quad \text{SNP associated with trait 1}) \end{aligned}$$

The conditional term in the right hand side of the equation above can be approximated as follows:

$$\begin{aligned} \mathbb{P}(\text{SNP associated with trait 2} \quad | \quad \text{SNP associated with trait 1}) &= \frac{p_{12}}{(p_{12} + p_1)} \\ &= \frac{10^{-6}}{(10^{-6} + 10^{-4})} \\ &\approx \frac{10^{-6}}{10^{-4}} \\ &\approx 0.01 \end{aligned}$$

So in terms of conditional probability, if we assume a prior probability of $p_{12} = 1 \times 10^{-6}$ and $p_1 = p_2 = 1 \times 10^{-4}$, then the prior assumption is that of all SNPs associated with trait 1, 1 in 100 of them will also be associated with trait 2. Since as we stated previously each SNP belongs to only one of the five sets corresponding to the five hypotheses, the probability of a SNP not being associated with either trait is: $p_0 = 1 - (p_1 + p_2 + p_{12}) \approx 0.9998 \approx 1$.

Choice of priors for the standard deviation W of the effect size parameter β

Prior standard deviation of the additive effect parameter β was set to 0.15 for a continuous trait. Owing to our assumption that $Var(Y) = 1$, this prior corresponds to a variance explained of ~ 0.01 for a variance with MAF of 30%.

In a case-control study, we set $W = 0.2$ for the variance of the log-odds ratio parameter, as was previously used in WTCCC [32]. The priors chosen for case-control and for quantitative traits are also very similar to the SNPTEST default ([17] and [53]).

Simulation procedure

To simulate the posterior probability of a common signal ("PP4") under different scenarios, we used the imputed genotypes from two different datasets: Whitehall II study (WHII), a longitudinal prospective cohort study genotyped using the gene-centric Illumina Metabochip [54, 55], and the expression dataset

described herein. We randomly chose a causal SNP A among genotyped and well imputed SNPs (Rsq > 0.8) from any genomic region in common between the two datasets.

The additive genetic variance explained by the locus is

$$Var[\beta X_A] = \beta_A^2 \times 2f_A(1 - f_A)$$

where f_A is the population allele frequency of the causal SNP A, and β_A is the additive effect (in standard deviations) [56].

For different variance explained for the causal SNP , depending on the simulation scenario, we computed the true effect at the causal SNP:

$$\beta_A = \sqrt{\frac{Var[\beta X_A]}{2f_A(1 - f_A)}}$$

We then simulated the phenotype of the ith individual in each of the two datasets using the computed true effect β_A:

$$Y_i = x_{iA} \times \beta_A + e_i \qquad with \quad e_i \sim N(0, 1)$$

where x_{iA} is the additive genetic value at the causal SNP of individual i and e_i is a random error drawn from a normal distribution with mean 0 and variance of 1.0.

To simulate p-values from different sample sizes using the original datasets, we computed the expected value of each estimated beta multiplying the pairwise correlation coefficient r between the causal SNP A and all other SNPs, by the true value of beta at the causal SNP:

The derivation of this equation is described in the following section.

The expected regression coefficient at a second SNP B in relation to the causal SNP A is then:

$$\beta_j = \beta_A \, r \sqrt{\frac{f_A(1 - f_A)}{f_j(1 - f_j)}} \,. \tag{4}$$

where f_j is a vector or βs for all SNPs excluding the causal SNP.

The difference between the regression coefficients $\hat{\beta}$ estimated from the glm and the expected value β, computed at each SNP, can be considered the standard error of the mean and it varies with $\frac{1}{\sqrt{(N)}}$, where N is the original sample size. When N is increased, the estimate becomes closer to the true value β, decreasing the variability of the estimator. Then the simulations with a larger sample size involves simply rescaling this difference:

$$(\hat{\beta} - \beta)\sqrt{\frac{N}{N.new}} \tag{5}$$

Then we can compute the new p-values from the new simulated estimates and standard deviations. We used this method to perform simulations to find sample size required for colocalisation analysis, and to find the consequence of using limited variant density.

Relationship between model parameters and LD

Equation 4 was first derived in [57], where the additive effects for case-control studies were shown to decay linearly, in proportion to r, the correlation between the causal and marker loci. Here we show the same relationship holds for quantitative traits.

We use the same LD model, and associated notation, as defined in [57]. To summarise briefly, let A and B be a pair of biallelic SNPs, with the alleles at each coded by 0 and 1. Let f_A be the population frequency of allele 1 at SNP A, and define f_B similarly for SNP B. Let r be the population correlation coefficient between them, the square of which is a commonly used measure of LD. We can define the following conditional probabilities on a haplotype level:

$$q_0 = P(A = 1 \mid B = 0)\,,$$
$$q_1 = P(A = 1 \mid B = 1)\,,$$

These quantities were shown to be related by the identity,

$$r = (q_1 - q_0)\sqrt{\frac{f_B(1-f_B)}{f_A(1-f_A)}}\,,$$

Let SNP A be a causal and SNP B be a marker. Define the following expectations:

$$\begin{aligned} a_0 &= \mathbb{E}(Y \mid A = 0)\,, & b_0 &= \mathbb{E}(Y \mid B = 0)\,, \\ a_1 &= \mathbb{E}(Y \mid A = 1)\,, & b_1 &= \mathbb{E}(Y \mid B = 1)\,, \\ a_2 &= \mathbb{E}(Y \mid A = 2)\,, & b_2 &= \mathbb{E}(Y \mid B = 2)\,. \end{aligned}$$

Relating these using the LD model gives,

$$\begin{aligned} b_0 &= a_0(1-q_0)^2 + a_1 2q_0(1-q_0) + a_2 q_0^2\,, \\ b_1 &= a_0(1-q_0)(1-q_1) + a_1\left(q_0(1-q_1) + q_1(1-q_0)\right) + a_2 q_0 q_1\,, \\ b_2 &= a_0(1-q_1)^2 + a_1 2q_1(1-q_1) + a_2 q_1^2\,. \end{aligned}$$

Combining and rearranging these gives,

$$b_1^2 - b_0 b_2 = \left(a_1^2 - a_0 a_2\right)(q_1 - q_0)^2\,.$$

If we consider an additive model, it is easy to show that $b_1^2 - b_0 b_2 = \beta_B^2$ and $a_1^2 - a_0 a_2 = \beta_A^2$. Putting these together gives,

$$\beta_B = \beta_A\, r\sqrt{\frac{f_A(1-f_A)}{f_B(1-f_B)}}\,. \tag{6}$$

Equation 6 is analogous to the respective results from [57] for binary traits. The main difference is that for quantitative traits here we have shown them to be exact. As before, we can see that the deviation effect decays more quickly with LD than does the additive effect: quadratically in r rather than linearly. This implies that the distortion effect described earlier for binary traits will extend also to quantitative traits.

Relationship between model parameters and LD using imputed genotypes

The previous section assumed the genotypes were observed without error. We can derive a similar relationship in the scenario where we instead use imputed genotype data.

Continuing with the same notation, we now allow A and B to have posterior distributions for each individual's genotype. The appropriate analysis should average the likelihood over these posteriors. A convenient approximation to this is to replace each genotype by its mean posterior value. [28] showed that this is a good approximation in the GWAS context. This leaves us in a similar situation as before, except that A and B have become continuous quantitative variables. The following general results apply for the regression parameters,

$$\beta_A = \frac{\operatorname{cov}(Y, A)}{\operatorname{var}(A)}\,,$$
$$\beta_B = \frac{\operatorname{cov}(Y, B)}{\operatorname{var}(B)}\,.$$

We need two assumptions to complete the derivation. Firstly, we require that Y and B are conditionally independent given A. In other words, $\operatorname{cov}(Y, B \mid A) = 0$. This is implicit in the definition of SNP A being the *causal* SNP, and is also implicitly assumed in the definitions in the previous section. The only extension here is that we assume this relationship still holds after imputation. This allows us to simplify the covariance of Y and B using the law of total covariance,

$$\begin{aligned}
\operatorname{cov}(Y, B) &= \mathbb{E}(\operatorname{cov}(Y, B \mid A)) + \operatorname{cov}(\mathbb{E}(Y \mid A), \mathbb{E}(B \mid A)) \\
&= \mathbb{E}(0) + \operatorname{cov}(\mathbb{E}(Y \mid A), \mathbb{E}(B \mid A)) \\
&= \operatorname{cov}(\mathbb{E}(Y \mid A), \mathbb{E}(B \mid A))\,.
\end{aligned}$$

The second assumption we need is that the conditional expectations of Y and B on A are both linear in A. That is,

$$\mathbb{E}(Y \mid A) = \theta_{Y0} + \theta_{Y1} A\,,$$
$$\mathbb{E}(B \mid A) = \theta_{B0} + \theta_{B1} A\,.$$

We already make this assumption for the relationship between Y and A, in using a linear regression model for the trait. The extra part here is that we assume the same type of relationship between the causal SNP and marker SNP. Since the underlying values for A and B are binary, this is reasonable.

Note that these are just standard linear regression equations, so we have that,

$$\theta_{Y1} = \frac{\operatorname{cov}(Y, A)}{\operatorname{var}(A)}\,,$$
$$\theta_{B1} = \frac{\operatorname{cov}(B, A)}{\operatorname{var}(A)}\,.$$

Putting all of these formulae together gives,

$$\begin{aligned}
\operatorname{cov}(Y, B) &= \operatorname{cov}(\mathbb{E}(Y \mid A), \mathbb{E}(B \mid A)) \\
&= \operatorname{cov}(\theta_{Y1} A, \theta_{B1} A) \\
&= \theta_{Y1}\theta_{B1} \operatorname{cov}(A, A) \\
&= \frac{\operatorname{cov}(Y, A)\operatorname{cov}(B, A)}{\operatorname{var}(A)}\,.
\end{aligned}$$

Dividing both sides by $\mathrm{var}(B)$ gives,

$$\begin{aligned}
\frac{\mathrm{cov}(Y,B)}{\mathrm{var}(B)} &= \frac{\mathrm{cov}(Y,A)}{\mathrm{var}(A)}\,\frac{\mathrm{cov}(B,A)}{\mathrm{var}(B)} \\
\frac{\mathrm{cov}(Y,B)}{\mathrm{var}(B)} &= \frac{\mathrm{cov}(Y,A)}{\mathrm{var}(A)}\,\frac{\mathrm{cov}(B,A)}{\sqrt{\mathrm{var}(A)\,\mathrm{var}(B)}}\,\sqrt{\frac{\mathrm{var}(A)}{\mathrm{var}(B)}} \\
\beta_B &= \beta_A\,\mathrm{cor}(B,A)\sqrt{\frac{\mathrm{var}(A)}{\mathrm{var}(B)}} \\
\beta_B &= \beta_A\,r\sqrt{\frac{\mathrm{var}(A)}{\mathrm{var}(B)}}\,.
\end{aligned}$$

This formula is analogous to equation (6). Note that if A and B are observed without error then we recover the previous formula.

Simulations to find sample size required for colocalisation analysis

We used the simulation method described above to compare the distribution of $PP4$ of randomly sampled regions under different scenarios. We used the original sample size of the eQTL dataset (n=966) and a variance explained of 10% for expression, while varying the sample size of the biomarker dataset and the proportion of the biomarker's variance explained by the causal variant.

Simulations to find consequence of using limited variant density

We compared the $PP4$ of randomly sampled regions, using the simulation procedure described above, with the $PP4$ of the same regions after filtering for only the SNPs present in the Illumina 660K Chip (Figures S1 and S2). The original dataset based on 1000 genomes imputed data will be much denser than the Illumina dataset. This way we can consider the consequences of having imputed data versus genotyped data. The same procedure is used to simulate the case when the causal SNP is not included in the data by excluding the causal SNP only from the Illumina dataset. All analyses were conducted in R [58].

Simulations for comparison with existing colocalisation tests and multivariate cases

For simulations comparing proportional and Bayesian approaches, we sampled, with replacement, haplotypes of SNPs with a minor allele frequency of at least 5% found in phased 1000 Genomes Project data [59] across all 49 genomic regions outside the major histocompatibility complex (MHC) which have been identified as type 1 diabetes (T1D) susceptibility loci to date, as summarised in T1DBase [60] (Figures S3 and S4). These represent a range of region sizes and genomic topography typical of GWAS hits. We excluded the MHC region which is known to have high variation, strong LD and exhibits huge genetic influence on autoimmune disease risk involving multiple loci and hence requires individual treatment in any GWAS [61]. For each trait, we selected one or two "causal variants" at random, and

simulated a Gaussian distributed quantitative trait for which each causal variant SNP explains a specified proportion of the variance. We either used all SNPs or the subset of SNPs which appear on the Illumina HumanOmniExpress genotyping array to conduct colocalisation testing to reflect the scenarios of very dense targeted genotyping *versus* a less dense GWAS chip. All analyses were conducted in R [58] using the `coloc` package for proportional colocalisation testing.

The same procedure is used to further explore multivariate cases in Figure S8.

Simulations for recessive model

For simulations under a recessive pattern of inheritance, we used the same simulation procedure as described in the previous paragraph, with the difference that after sampling haplotypes we recoded the major homozygous and heterozygous to 0 and minor homozygous to 1 (Figure S9).

References

52. McCullagh P, Nelder J (1983) Generalized Linear Models, Chapman & Hall, chapter 4.
53. Marchini J, Howie B, Myers S, McVean G, Donnelly P (2007) A new multipoint method for genome-wide association studies by imputation of genotypes. Nature genetics 39: 906–913.
54. Voight BF, Kang HM, Ding J, Palmer CD, Sidore C, et al. (2012) The metabochip, a custom genotyping array for genetic studies of metabolic, cardiovascular, and anthropometric traits. PLoS genetics 8: e1002793.
55. Talmud PJ, Drenos F, Shah S, Shah T, Palmen J, et al. (2009) Gene-centric association signals for lipids and apolipoproteins identified via the humancvd beadchip. American journal of human genetics 85: 628.
56. D S Falconer DS, Mackay TFC (1996) Introduction to Quantitative Genetics. Longman Pub Group.
57. Vukcevic D, Hechter E, Spencer C, Donnelly P (2011) Disease model distortion in association studies. Genetic epidemiology 35: 278–290.
58. Team RDC (2013) R: A Language and Environment for Statistical Computing. R Foundation for Statistical Computing, Vienna, Austria. URL `http://www.R-project.org`.
59. Autosomes Chromosome X (2012) An integrated map of genetic variation from 1,092 human genomes. Nature 491: 1.
60. Burren OS, Adlem EC, Achuthan P, Christensen M, Coulson RM, et al. (2011) T1dbase: update 2011, organization and presentation of large-scale data sets for type 1 diabetes research. Nucleic acids research 39: D997–D1001.
61. Nejentsev S, Howson JM, Walker NM, Szeszko J, Field SF, et al. (2007) Localization of type 1 diabetes susceptibility to the mhc class i genes hla-b and hla-a. Nature 450: 887–892.
