# Supplementary material for: Bayesian Test for Colocalisation between Pairs of Genetic Association Studies Using Summary Statistics
Source: PLoS Genet. 2014 May 15;10(5):e1004383. doi: 10.1371/journal.pgen.1004383 (PMC4022491; doi:10.1371/journal.pgen.1004383)
Supplement: Text S2 — Overview of gene function of new colocalisation results associated with blood lipid levels and liver expression. (PDF) [file pgen.1004383.s016.pdf]

# Overview of gene function of new colocalisation results associated with blood lipid levels and liver expression

- *SDC1* (Syndecan-1) encodes a transmembrane heparan sulfate proteoglycan which mediates the clearance of triglyceride-rich lipoproteins in liver [42].
- *TGOLN2* (Trans-Golgi Network Protein 2) encoded by this gene regulates cholesterol transport to the trans-Golgi network and plasma membrane caveolae [43].
- *INHBB* encodes Inhibin beta B, a subunit of both activin and inhibin. This locus has been reported associated with elevated levels of lipids and cardiovascular disease risk traits [44].
- *UBXN2B* (UBX Domain Protein 2B) encodes a protein containing a UBX-domain involved in endoplasmic reticulum-associated degradation, a process which is crucial for lipid droplets maintenance [45].
- *VLDLR* (Very Low Density Lipoprotein Receptor) is known to play important roles in VLDL-triglyceride metabolism, and has been previously correlated with glucose and triglyceride plasma levels [46].
- *VIM* codes for Vimentin, and a functional role for vimentin intermediate filaments has been reported in the metabolism of lipoprotein-derived cholesterol [47].
- *CYP26A1* encodes an endoplasmic reticulum protein which regulates the the cellular level of retinoic acid, a critical signalling molecule involved in the regulation of gene expression. This protein belongs to the cytochrome P450 superfamily of enzymes that catalyse many reactions involved in maintenance of lipid homeostasis and drug metabolism [48].
- *OGFOD1* (2-oxoglutarate and iron-dependent oxygenase domain containing 1) is crucial for cellular adaptation to changes in oxygen concentration, and has been reported to function in ischaemic signalling [49].
- *HP* encodes the plasma protein haptoglobin, which binds and transports free haemoglobin (Hb) released from erythrocytes back to the liver for recycling, thereby inhibiting haemoglobin's oxidative activity [50].
- Haptoglobin-related protein (*HPR*) is a plasma protein associated with apolipoprotein-L-I containing high-density lipoprotein (HDL) particles, and has been shown to be part of the innate immune response [51].

  *HP* and *HPR* have been previously associated with lipids [62].
- *PPARA* gene encodes the transcription factor peroxisome proliferator-activated receptor alpha (PPAR-alpha), a major regulator of lipid metabolism in the liver. PPAR-alpha serves as cellular receptor for fibrates, an anti-dyslipidaemia d drug that effectively lower serum triglycerides and raise serum HDL-cholesterol levels [52].

# References

62. Guthrie PA, Rodriguez S, Gaunt TR, Lawlor DA, Smith GD, et al. (2012) Complexity of a complex trait locus: Hp, hpr, haemoglobin and cholesterol. Gene 499: 8–13.
